# Epidemiology of 369 diseases and injuries attributable to 84 risk factors: 1990–2019 with 2040 projection

**DOI:** 10.1016/j.isci.2024.109508

**Published:** 2024-03-14

**Authors:** Kexin Zhang, Chengxia Kan, Jian Chen, Junfeng Shi, Yanhui Ma, Xiaoli Wang, Xuan Li, Weiqin Cai, Ruiyan Pan, Jingwen Zhang, Zhentao Guo, Fang Han, Ningning Hou, Xiaodong Sun

**Affiliations:** 1 Department of Endocrinology and Metabolism, Clinical Research Center, Affiliated Hospital of Shandong Second Medical University, Weifang, China; 2 School of Basic Medical Sciences, Guilin Medical University, Guilin, China; 3 Department of Pathology, Affiliated Hospital of Shandong Second Medical University, Weifang, China; 4 School of Medical Imaging, Weifang Medical University, Weifang, China; 5 Department of Physiology and Biophysics, University of Mississippi Medical Center, Jackson, MS, USA; 6 School of Management, Weifang Medical University, Weifang, China; 7 Department of Pharmacy, Weifang Medical University, Weifang, China; 8 Department of Nephrology, Affiliated Hospital of Shandong Second Medical University, Weifang, China

**Keywords:** Health sciences, Medical specialty, Public health, Natural sciences, Environmental science, Pollution

## Abstract

The global burden of diseases and injuries poses complex and pressing challenges. This study analyzed 369 diseases and injuries attributed to 84 risk factors globally from 1990 to 2019, projecting trends to 2040. In 2019, global risks caused 35 million deaths. Non-communicable diseases were responsible for 8.2 million deaths, primarily from air pollution (5.5 million). Cardiovascular disease from air pollution had a high age-standardized disability-adjusted life year rate (1,073.40). Communicable, maternal, neonatal, and nutritional diseases caused 1.4 million deaths, mainly due to unsafe water and sanitation. Occupational risks resulted in 184,269 transport-related deaths. Behavioral risks caused 21.6 million deaths, with dietary factors causing 6.9 million cardiovascular deaths. Diabetes linked to sugar-sweetened beverages showed significant growth (1990–2019). Metabolic risks led to 18.6 million deaths. Projections to 2040 indicated persistent challenges, emphasizing the urgent need for targeted interventions and policies to alleviate the global burden of diseases and injuries.

## Highlights

• Upward trend in deaths from behavioral and metabolic risks (1990–2019)
• Environmental/occupational risks persist as significant global contributors
• Metabolic risks drive NCD deaths; behavioral risks lead CMNND fatalities
• Projected rise in NCD deaths from environmental/occupational and behavioral risks

## Graphical abstract

**Figure:**
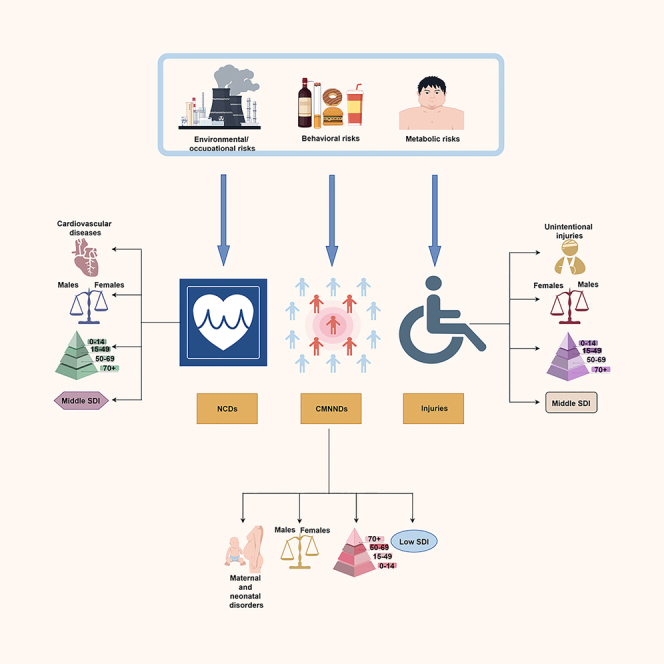

## Introduction

The global burden of diseases and injuries represents a multifaceted and pressing issue that significantly impacts individuals, communities, and societies.^1^ It encompasses a range of health conditions, including non-communicable diseases (NCDs) and communicable, maternal, neonatal, and nutritional diseases (CMNNDs) and injuries.^1^^,^^2^^,^^3^ Effectively addressing this issue requires a thorough understanding of the specific contributing risk factors. During the past three decades, the global health landscape has undergone transformative changes in demographics, socioeconomic factors, and the prevalence of various risk factors.^4^^,^^5^^,^^6^ These changes, coupled with advancements in data collection and analytical methodologies, present an opportune moment to assess the global burden of diseases and injuries and its association with specific risk factors.

Environmental/occupational, behavioral, and metabolic risks emerge as primary contributors to health impairment, exerting profound effects on both disease incidence and injury occurrence.^5^ Implementing interventions targeting these factors at a population level holds the potential for yielding substantial reductions in the burden of disease. The Global Burden of Disease (GBD) Study 2019 collected and furnished an exhaustive analysis and estimates of diverse health conditions across 204 countries from 1990 to 2019.^1^ However, these estimates focused on specific diseases without conducting risk factors analysis or only examined individual risk factors with limited geographic coverage, thereby impeding a comprehensive understanding of their overall impact and regional variations in global health.

Thus, our objective is to provide a comprehensive overview of 369 diseases and injuries attributable to risk factors worldwide from 1990 to 2019. Through the analysis of the intricate interplay between these risk factors and diseases or injuries, our study endeavors to gain a comprehensive understanding of the global health landscape while forecasting the global burden through 2040 and identifying priority areas requiring intervention. The findings from this research possess profound implications for global health, offering valuable insights to guide evidence-based interventions and strategies that alleviate the burden of diseases and injuries worldwide.

## Results

### Environmental/occupational risks

#### Global analysis

**Figure 1:**
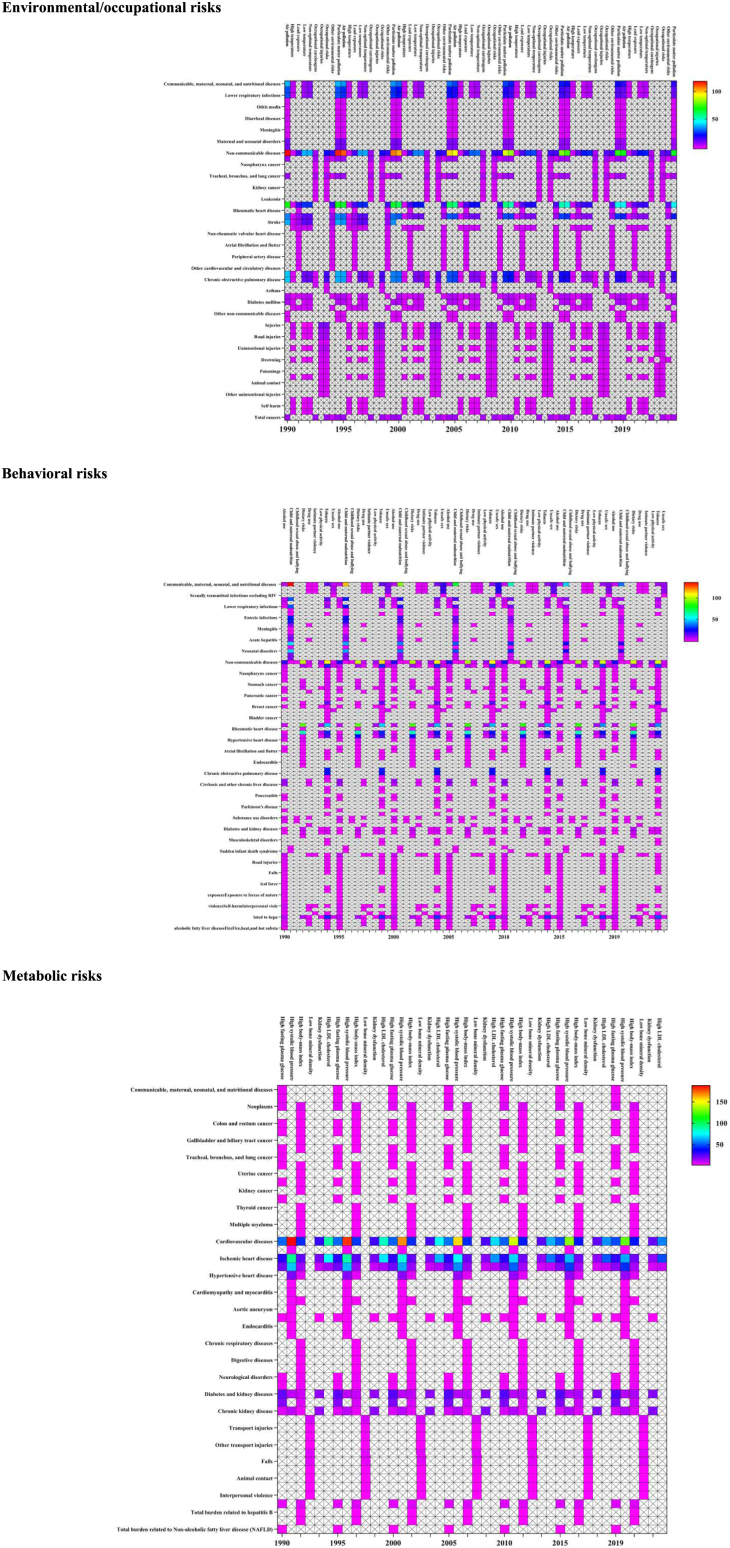
ASMR of NCDs, CMNNDs, and injuries attributable to different environmental/occupational risks, behavioral risks, and metabolic risk factors, 1990–2019 See also Figures S1–S3; Tables S1 and S2.

Globally, there were 11.3 million (10.3–12.4) total deaths caused by environmental/occupational risks in 2019 (Figures S1 and S2, and Table S1). Among NCDs, air pollution was the leading cause of death, accounting for 5.5 million (4.9–6.2) deaths with age-standardized mortality rate (ASMR) of 69.3 (60.9–77.7). These deaths were primarily caused by cardiovascular diseases (CVDs; ischemic heart disease [IHD] and stroke), chronic respiratory diseases, neoplasms (tracheal, bronchus, and lung cancer), type 2 diabetes, and sudden infant death syndrome. Interestingly, air pollution (particulate matter [PM] pollution) predominantly contributes to deaths from IHD in men (1,080,097 [932,622–1,238,248]) and stroke in women (780,080 [671,813–901,537]). Additionally, non-optimal temperature emerged as a closely linked factor to deaths, ranking second to air pollution, with a total of 1.8 million (1.5–2.0) deaths. The primary outcomes of this association were observed in CVD, specifically IHD and stroke, accounting for 596,780 (414,250–763,263) deaths and 521,031 (402,433–663,996) deaths, respectively. Notably, the number of deaths from "chronic kidney disease due to other and unspecified causes" caused by high temperature increased 67.8 times from 1990 to 2019. Among different age groups, global trends in NCDs reveal distinct patterns. In the age group of 0–14, diabetes and kidney disease due to non-optimal temperature were the main causes of NCD-related deaths (1,011 [121–1,712] cases). For individuals aged 15 and above, CVD resulting from air pollution was the predominant cause (Figures 1, S2, and S3, and Table S2).

In 2019, there were 220,062,261 (197,905,935–241,015,722) cases of disability-adjusted life years (DALYs) for NCDs due to environmental factors. The NCDs (141,234,492 [124,618,269–157,101,097]) were mainly attributed to air pollution with a corresponding age-standardized DALY rate (ASDR) of 1,713.79 (1,512.23–1,904.44). The major NCDs were CVD, chronic respiratory diseases, and type 2 diabetes. The ASDR of CVD due to air pollution was as high as 1,073.40 (956.99–1,195.14). From 1990 to 2019, the number of DALYs cases for chronic respiratory diseases caused by high temperatures increased by up to 16.16 times. The ASDR for chronic respiratory diseases caused by high temperatures increased the most (estimated annual percentage change [EAPC], 6.06 [95% confidence interval, CI, −6.62 to 6.79]). Both men and women exhibited the same trend, with both having the highest number of DALYs and the highest ASDR for CVD caused by air pollution.

Among CMNNDs, the ASMR for these diseases was 39.7 (32.8–48.5). Enteric infections, such as diarrheal diseases, were primarily caused by unsafe water, sanitation, and handwashing, resulting in 1.4 million (1.0–2.0) deaths. Air pollution, particularly PM pollution, was the second leading cause of CMNNDs-related deaths, with 749,254 (573,848–959,290) deaths attributed to respiratory infections and tuberculosis. Neonatal sepsis and other neonatal infections related to ambient PM pollution also increased by 0.71-fold (0.16–1.73) from 1990 to 2019. Enteric infections related to unsafe water, sanitation, and handwashing were prominent causes of death in all CMNNDs age groups, with the highest number of deaths occurring in the 0–14 age group (519,868 [403,847–666,324]). In terms of injuries, occupational risks accounted for the highest number of deaths from transport injuries (184,269 [166,416–205,855]). High temperatures were responsible for deaths caused by self-harm and interpersonal violence (26,459 [13,574–47,265] cases). Deaths resulting from motorcyclist road injuries due to high temperatures witnessed a 0.72-fold (0.03–1.56) increase from 1990 to 2019. Unintentional injuries caused by high temperature were the primary cause in children aged 0–14 (4,787 [2,116–8,417] cases). Deaths from transport injuries due to occupational risks were highest in 15–69 years (15–49 years: 146,555 [132,802–162,286] cases, 50–69 years: 35,826 [31,615–41,421] cases). Unintentional injuries were notable among those over 70 years (3,349 [2,892–3,831] cases) (Figures 1 and S3, and Table S2).

#### Analysis from sociodemographic index (SDI) regions

**Figure 2:**
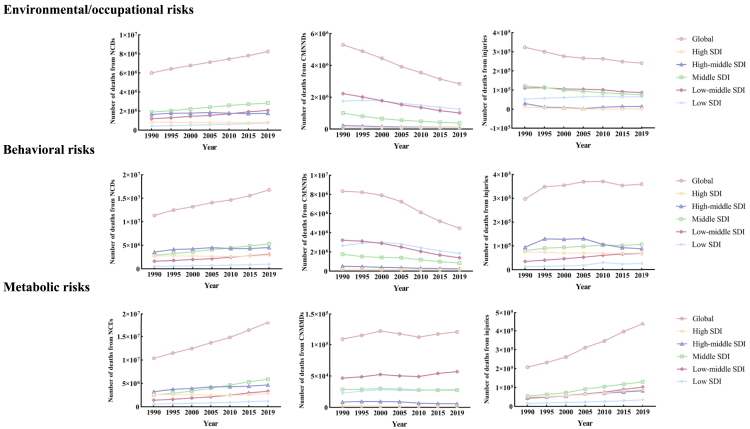
In the five SDI regions, environmental/occupational risks, behavioral risks, and metabolic risks contribute to the number of deaths from NCDs, CMNNDs, and injuries

Deaths from environmental/occupational risks among NCDs varied across SDI regions (Figure 2). The low-SDI and low-middle-SDI region experienced the highest increase in NCDs-related deaths (0.72-fold, [0.50–0.94]; 0.72-fold, [0.51–0.93], respectively) from 1990 to 2019, while the low-middle-SDI region had the highest ASMR (170.2 [149.3–189.9]). Among the five SDI regions, the low-SDI region had the highest ASDR of 4,288.37 (3,793.90–4,761.98) for NCDs due to environmental/occupational risks. Specifically, IHD caused by PM pollution was predominant in the low-SDI region, with an ASDR of 940.68 (805.44–1,093.68). However, its ASDR decreased from 1990 to 2019, with an EAPC of −0.22 (95% CI, −0.35 to −0.08). During the same period, the low-middle-SDI region saw the greatest increase in ASDR for chronic obstructive pulmonary disease caused by high temperatures.

In CMNNDs, the low-SDI region had the highest ASMR in 2019 (149.9 [120.0–190.6]), primarily due to diarrheal diseases caused by unsafe water, sanitation, and handwashing. The changes in deaths varied among SDI regions, with rising trends in the high-SDI region and decreasing trends in other regions, particularly the middle-SDI region. In terms of injuries, the low-SDI region had the highest increase (0.23-fold [0.05–0.44]) in deaths, while the other regions exhibited a decreasing trend, with the high-SDI region having the largest decrease.

#### Analysis from the GBD regions

In 2019, East Asia, South Asia, Southeast Asia, and North Africa/Middle East had the highest number of deaths from environmental/occupational risks in terms of NCDs, while Oceania had the highest ASMR (212.6 [167.5–263.0]). In East Asia, CVDs caused by air pollution (PM pollution) were the predominant cause of NCDs-related deaths. In 2019, East Asia had the highest number of DALYs cases (63,821,268 [55,298,943–72,423,324]). Most deaths were due to CVD primarily caused by PM pollution (27,075,620 [23,016,028–31,462,143]), resulting in a high ASDR of 1,327.18 (1,132.71–1,539.91). From 1990 to 2019, Eastern Europe witnessed the largest increase in ASDR for hypertensive heart disease due to high temperatures, with an EAPC of 24.27 (−33.46 to 47.46).

**Figure 3:**
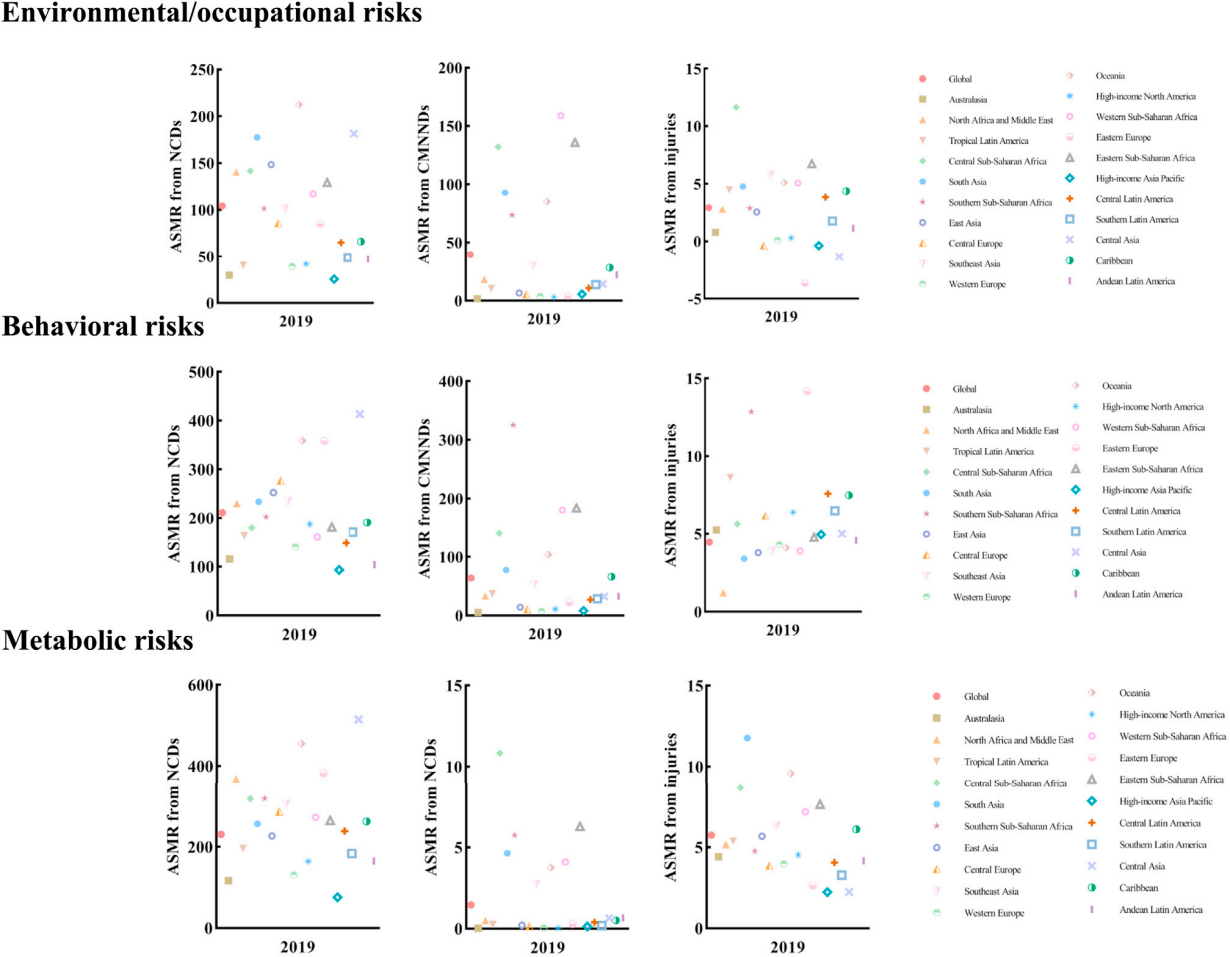
In 21 geographic areas, environmental/occupational risks, behavioral risks, and metabolic risks contribute to the number of deaths in NCDs, CMNNDs, and injuries

Regarding CMNNDs, South Asia, Western Sub-Saharan Africa, and Eastern Sub-Saharan Africa had the highest number of deaths, with Western Sub-Saharan Africa having the highest ASMR (159.0 [127.2–199.0]). South Asia and Western Sub-Saharan Africa were particularly affected by high numbers of deaths from enteric infections caused by unsafe water, sanitation, and handwashing practices. In terms of injuries, South Asia, Southeast Asia, and East Asia had the highest number of deaths from environmental/occupational risks (Figure 3).

#### Analysis from 204 countries

**Figure 4:**
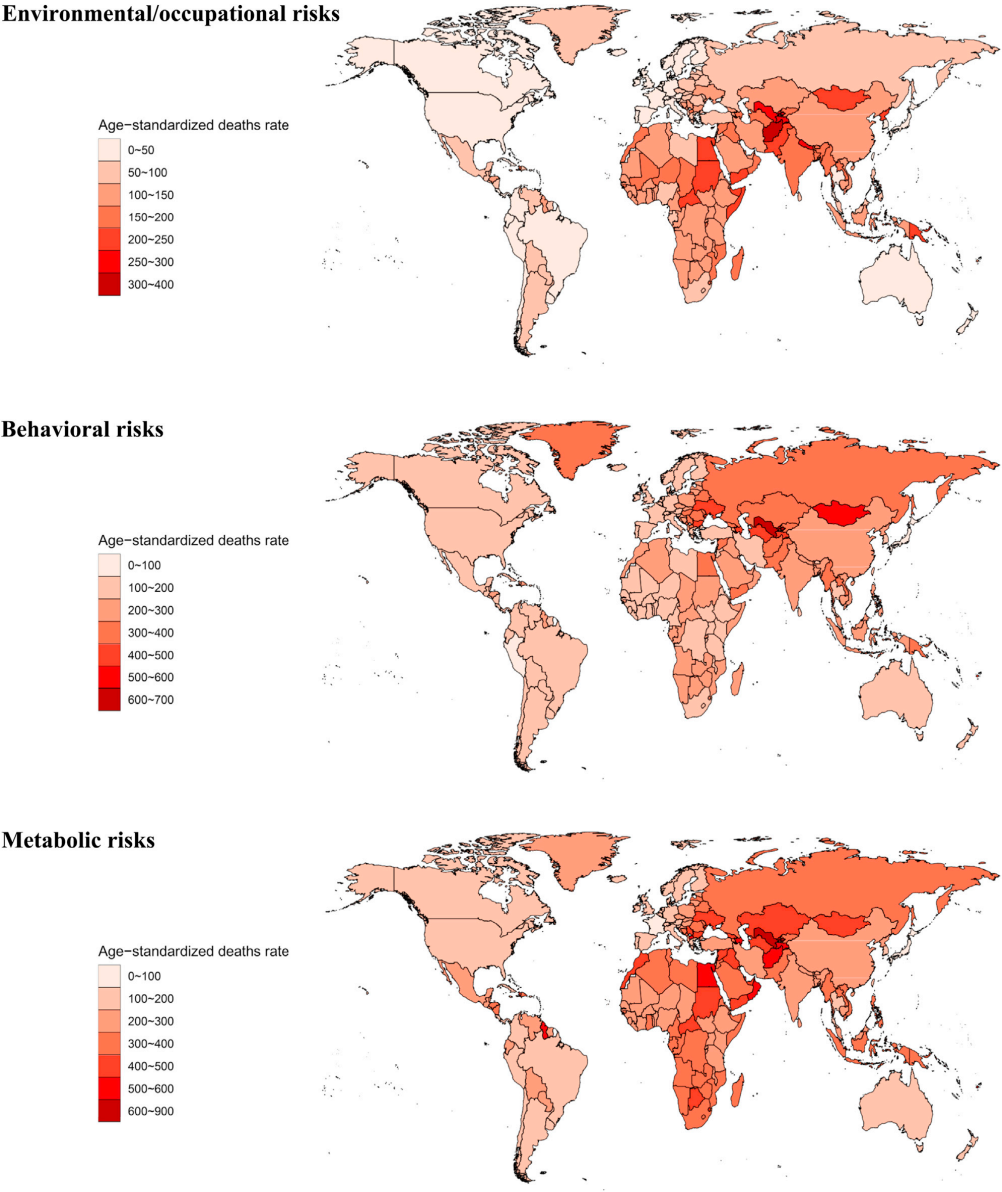
ASMR of non-communicable diseases due to environmental/occupational risks, behavioral risks, and metabolic risks in 204 countries See also Figures S4 and S5.

Among the 204 countries, China and India had the highest number of deaths from NCDs caused by environmental/occupational risks, with 2.6 million (2.2–3.0) and 1.8 (1.5–2.0) deaths, respectively (Figure 4). In contrast, the countries with the highest ASMR for NCDs were Solomon Islands, Afghanistan, and Uzbekistan, with ASMRs of 392.4 (317.3–470.6), 308.2 (244.8–370.9), and 294.9 (233.0–362.5), respectively. In 2019, the highest number of DALYs cases for NCDs due to environmental/occupational risks occurred in China and India. China had the highest number of cases of CVD caused by PM pollution (26,129,320 [22,136,925–30,533,160]) with an ASDR of 1,328.87. Additionally, China had the highest number of DALYs cases resulting in death due to PM pollution (26,129,320 [22,136,925–30,533,160]) with an ASDR of 1,328.87 (1,130.76–1,546.54).

For CMNNDs, India, Nigeria, and Pakistan had the highest number of deaths, while the Central African Republic exhibited the highest ASMR of 341.2 (239.7–472.7). In terms of injuries, India, China, and Indonesia recorded the highest number of injuries. Somalia has the highest ASMR of 20.3 (16.5–25.3) (Figures S4 and S5).

### Behavioral risks

#### Global analysis

In 2019, behavioral risks globally caused 21.6 million (20.0–23.4) deaths with distinct patterns among different age groups (Table S1, Figures S1 and S2). Worldwide, the ASMR for deaths caused by NCDs resulting from behavioral risks was 211.5 (193.4–233.1). Tobacco, dietary risks, and alcohol use were the primary behavioral factors contributing to these diseases. Smoking contributed to a substantial number of deaths from CVD, neoplasms, and chronic respiratory diseases, totaling 2.7 million (2.5–2.9) deaths, 2.5 million (2.3–2.7) deaths, and 1.6 million (1.5–1.8) deaths, respectively. The number of deaths from CVD caused by dietary risk was (6.9 million [5.6–8.4]) with the corresponding ASMR of 87.4 (70.6–107.4). Dietary risks associated with CVD mainly included high sodium intake and low consumption of whole grains, resulting in 1.7 million (0.5–3.7) deaths and 1.6 million (0.7–2.1) deaths, respectively. Deaths from intracerebral hemorrhage were linked to diets high in sodium, low in fruits, and high in red meat, resulting in 371,328 deaths (111,629–763,628), 237,918 deaths (130,217–395,463), and 192,341 deaths (91,602–278,126), respectively. Colon and rectum cancer deaths were associated with diets low in whole grains, low in milk, and low in calcium, resulting in 171,487 deaths (66,721–225,162), 166,456 deaths (107,221–226,027), and 137,896 deaths (96,835-189,221), respectively. Further, alcohol use was a significant risk factor for death from cirrhosis and other chronic liver diseases, resulting in 712,815 deaths (564,602–876,150). CVDs were primarily caused by dietary risks and tobacco in both sexes, while, among females, diet plays a dominant role. Deaths from cocaine use disorder due to drug use had significantly increased by 4.43-fold (3.52–4.89) from 1990 to 2019. In terms of NCDs, deaths among individuals aged 0–14 years were primarily attributed to sudden infant death syndrome associated with low birth weight and short gestation, resulting in 1,942 deaths (1,076–3,740). Among those aged 15 years and older, deaths from CVD were mainly caused by dietary risks (Figures S3 and S6).

In 2019, there were 454,488,490 DALYs associated with NCDs due to behavioral risks, primarily stemming from dietary risks and tobacco. The number of DALYs for CVD, along with their corresponding ASDRs, were as follows: dietary risks (ASDR: 1,870.06 [1,533.60–2,272.81]) and tobacco (ASDR: 993.42 [919.78–1,071.48]). From 1990 to 2019, diabetes mellitus caused by a diet high in sugar-sweetened beverages showed the most significant increase in ASDR (EAPC: 0.32 [95% CI, 0.15–0.43]). Both sexes exhibited the same trend, having the highest ASMR for dietary risks leading to CVD.

In terms of CMNNDs, the ASMR due to behavioral risks was 63.7 (56.2–73.1). The main causes of death included child and maternal malnutrition, unsafe sex, tobacco, and alcohol use. Child and maternal malnutrition mainly causes neonatal diseases resulting in 1.6 million (1.4–1.9) deaths mainly due to low birth weight and short gestation. Child growth failure causes enteric infections deaths (405,102 [311, 396–528,491]) and protein-energy malnutrition-related deaths (212,242 [185,403–246,217]). Unsafe sex led to deaths primarily from HIV/AIDS (acquired immunodeficiency syndrome) and sexually transmitted infections, totaling 703,887 (636,225–819,617) deaths. Smoking mainly resulted in respiratory infections and tuberculosis deaths, with 457,929 (375,058–545,739) deaths. Alcohol use primarily contributes to deaths from respiratory infections and tuberculosis, accounting for 292,427 (206,988–374,510) deaths. Neonatal disorders due to low birth weight and short gestation significantly affected both sexes. HIV/AIDS-multidrug-resistant tuberculosis without extensive drug resistance caused by unsafe sex exhibited the highest growth rate of 33.76-fold (18.28–59.78) from 1990 to 2019. Among different age groups, deaths among individuals aged 0–14 years were primarily caused by neonatal diseases associated with low birth weight, resulting in 1.5 million (1.3–1.8) deaths. Among those aged 15–49 years, deaths from HIV/AIDS and sexually transmitted infections were mainly attributed to unsafe sex, totaling 515,236 (431,039–642,598) deaths. For individuals aged 50 years and older, deaths from respiratory infections and tuberculosis were predominantly caused by tobacco use (50–69 years: 187,812 [159,604–218,779] deaths; 70 years and older: 284,504 deaths [227,883–343,586]) (Table S1; Figures S3 and S6).

The global ASMR for injuries caused by behavioral risks was 4.5 (3.5–5.5) with alcohol use, tobacco, intimate partner violence, and drug use being key contributors. Alcohol use was linked to deaths from self-harm and interpersonal violence, resulting in 160,442 (106,475–218,053) deaths. Tobacco, specifically smoking, primarily led to deaths from falls, totaling 21,069 (14,885–28,394) deaths. Intimate partner violence was a major cause of deaths related to interpersonal violence, accounting for 21,506 (15,223–28,777) deaths. Self-harm deaths in males were primarily associated with alcohol use, resulting in 100,497 (53,675–149,575) deaths. Interpersonal violence deaths in females were mainly attributed to intimate partner violence, resulting in 21,506 (15,223–28,777) deaths. From 1990 to 2019, tobacco resulted in a 0.93-fold (0.62–1.23) increase in deaths from falls. Among individuals aged 0–14 years, deaths from transportation injuries were primarily caused by alcohol use, resulting in 2,526 (1,494–3,662) deaths. Among those aged 15–69 years, deaths from self-inflicted injuries and interpersonal violence were primarily linked to alcohol use. For individuals aged 70 years and older, deaths from falls were primarily attributed to alcohol use, totaling 17,060 (8,162–29,291) deaths (Table S1; Figures S3 and S6).

#### Analysis from SDI regions

Behavioral risks in NCDs varied across SDI regions, with the most significant increase observed in the low-middle-SDI region, which experienced a 0.90-fold increase (0.73–1.08) from 1990 to 2019 (Figure 2). The highest ASMR in the region was 246.0 (220.1–274.7). Middle-SDI regions had notable deaths from dietary risks (diets high in sodium) and tobacco-related CVD. High-SDI regions saw a remarkable increase in deaths from cocaine use disorders, with an 11.08-fold (10.04–12.02) increase. Of the five SDI regions, the middle-SDI region had the highest number of DALYs with NCDs due to behavioral risks (143,671,618 [128,948,097–159,767,988]); specifically, this mainly includes cases of neoplasms DALYs due to tobacco and CVD due to diet high in sodium. In the low-middle-SDI region, IHD due to dietary risks has the highest ASDR.

In CMNNDs, all SDI regions experienced a decline, with the largest decrease observed in the low-middle-SDI region (−0.57-fold [−0.63 to −0.51]). The highest ASMR in the low-SDI region was 140.0 (122.6–161.8). Neonatal disorders caused by low birth weight and short gestation had the highest number of deaths in the low-SDI region, totaling 713,452 (590,012–872,511). Surprisingly, the middle-SDI region experienced a significant 99.40-fold increase (30.84–347.99) in deaths from HIV/AIDS-multidrug-resistant tuberculosis without extensive drug resistance due to intimate partner violence. Injuries related to behavioral risks varied across SDI regions, with the low-SDI region showing the most substantial increase (1.05 [0.77 to 1.40]). However, the region with the highest ASMR was the high-SDI region (5.2 [4.0–6.4]). Middle-SDI regions had the highest number of deaths from self-harm and interpersonal violence caused by alcohol use, totaling 42,950 (28,784–57,448). Exposure to forces of nature due to alcohol use increased 4.01-fold (2.82–5.22) in deaths in low-SDI regions.

#### Analysis from GBD regions

Within the GBD region, East Asia and South Asia had high rankings for deaths from CVD due to dietary risks, with 1.8 million (1.3–2.4) deaths and 1.3 million (1.1–1.6) deaths, respectively. South Asia also saw significant increases in type 2 diabetes due to alcohol use (31.5 [−41.7 to 45.7]). The region with the highest ASMR was Central Asia, with a rate of 413.0 (367.6–461.7). In high-income North America, there were rises in deaths from amphetamine and cocaine use disorders due to drug use, with rates of 17.53 (15.37–20.42) and 13.12 (11.82–14.34), respectively. In 2019, Eastern Europe had the highest ASDR for NCDs due to behavioral risks (9,739.62 [8,702.52–10,863.50]). Specifically, the ASDR for IHD, mainly attributable to a diet low in whole grains, was the highest (985.46 [334.80–1,296.48]), resulting in a substantial number of DALYs (3,279,052 [1,109,908–4,316,797]). Between 1990 and 2019, Southeast Asia experienced the most significant increase in ASDR for CVD caused by alcohol use.

Among CMNNDs, South Asia and Western Sub-Saharan Africa had the highest number of deaths from neonatal disorders caused by low birth weight and short gestation. Southern Sub-Saharan Africa had the highest ASMR, with a rate of 325.2 (294.7–369.2). Oceania witnessed a significant increase in the number of deaths due to unsafe sex in HIV/AIDS-multidrug-resistant tuberculosis without extensive drug resistance. Injuries in Eastern Europe were notable for self-harm and interpersonal violence deaths associated with alcohol use, with an ASMR of 8.36 (5.64–11.39). East Asia recorded the highest number of deaths from transport injuries due to alcohol use, totaling 20,256 (11,643–30,009). Southern Sub-Saharan Africa experienced significant increases in deaths caused by exposure to forces of nature due to alcohol use, with increases of 16.39-fold (12.46–20.16) (Figure 3).

#### Analysis from 204 countries

Among the GBD regions, China, India, the USA, and Russia had high behavioral risks contributing to NCDs, especially CVDs. China’s primary risk factor was tobacco (57.42%), while dietary risks were significant in India (49.69%). Solomon Islands had the highest ASMR of 656.5 (538.7–771.0) due to NCDs deaths (Figure 4). India had the highest number of deaths from CMNNDs. Lesotho had the highest ASMR for CMNNDs (733.8 [643.9–852.9]) with HIV/AIDS mainly caused by unsafe sex, corresponding to an ASMR of 537.7 (460.8–661.9). Injuries resulted in the highest number of deaths in China, India, the USA, and the Russian Federation. Alcohol use was a significant risk factor in India (76.04%). Lesotho, Guyana, and Greenland had the highest ASMR for injuries, with Lesotho having the highest ASMR of 19.3 (11.6–28.3) (Figures S4 and S5). In 2019, China and India had the highest number of cases of NCDs DALYs due to behavioral risks, with China primarily attributed to tobacco and India primarily linked to dietary risks. Solomon Islands had the highest number of NCDs due to behavioral risks, with an ASDR of 17,959.52 (14,667.99–21,404.46), where tobacco and a diet low in whole grains were major contributors to IHD. From 1990 to 2019, Albania had the largest increase in ASDR for CVD caused by alcohol use.

### Metabolic risks

#### Global analysis

Globally, in 2019, metabolic risks caused 18.6 million (16.9–20.2) deaths, primarily from NCDs, with distinct patterns observed among different age groups (Figures S1 and S2, and Table S1). High systolic blood pressure (SBP) was the leading metabolic factor, accounting for 39.8% of NCDs deaths (10.8 million [9.5–12.1]). CVDs, particularly IHD, were the primary cause of deaths related to high SBP, totaling 4.9 million (3.9–5.9) deaths. In the 0–14 age group, there were 16,946 deaths (14,390–19,828) attributed to chronic kidney disease (CKD), primarily resulting from renal dysfunction. In individuals aged 15 years and older, CVD primarily caused by high SBP accounted for a substantial number of deaths: 642,039 deaths (539,261–749,029) in the 15–49 years group, 3,104,331 deaths (2,751,398–3,431,366) in the 50–69 years group, and 6,219,604 deaths (5,212,013–7,215,296) in the 70 years and older group. In 2019, the world experienced a significant number of DALYs due to metabolic risks resulting in NCDs (442,351,635 [401,428,391–486,992,361]). The majority of these cases were fatal DALYs attributed to CVD, primarily due to high SBP (213,915,370 [190,687,598–237,675,856]), with corresponding ASDR of 2,621.16 [2,339.38–2,920.64]). From 1990 to 2019, the greatest increase in ASDR was observed in diabetes mellitus due to high body mass index (EAPC: 0.83 [95% CI, 0.59 to 1.18]). Both sexes exhibited high ASDR in CVD caused by high SBP.

Among CMNNDs, respiratory infections and tuberculosis caused by high fasting plasma glucose (FPG) were responsible for the highest number of deaths, totaling 120,372 (75,828–171,928) deaths (81,708 [50,463–116,223] deaths in males and 38,665 [23,007–57,585] deaths in females) with the corresponding ASMR of 1.46 (0.91–2.08). Falls associated with low bone mineral density (BMD) accounted for 68.85% of injury deaths. The mortality rate from falls related to low BMD showed substantial growth from 1990 (121,248 deaths) to 2019 (301,482 deaths). In individuals aged 15 and older, respiratory infections and tuberculosis caused by high FPG were the main contributors to mortality. Regarding injuries, in individuals aged 15–69 years, traffic injuries caused by low BMD accounted for the majority of deaths (Figures S3 and S7).

#### Analysis from SDI regions

The middle-SDI region had the highest number of cases of NCDs caused by metabolic risk factors. The low-middle-SDI region experienced the largest increase (1.36-fold [1.15–1.56]) in deaths, with the highest ASMR of 273.9 (246.7–301.4) in 2019. CVD caused by high SBP resulted in the highest number of deaths in middle-SDI region, totaling 3.3 million (2.9–3.7), and pancreatic cancer caused by high body mass index showed the fastest increase (8.66 [6.27–13.35]) in low-middle SDI. Among CMNNDs, the highest number of deaths attributable to metabolic risk factors was in the low-middle-SDI region. Among the five SDI regions, the middle-SDI region had the highest number of DALYs cases with NCDs due to metabolic risk (151,293,128 [136,984,098–166,823,237]), predominantly high SBP leading to CVD in cases of DALYs (74,415,575 [65,941,453–83,191,262]). The middle-SDI region has the highest ASDR for IHD due to high SBP (1,341.37 [1,124.31–1,573.73]). From 1990 to 2019, middle SDI had the greatest increase in ASDRs by high body mass index leading to pancreatic cancer with a corresponding EAPC of 1.82 (1.33–2.66).

In CMNNDs, the low-SDI region experienced a significant increase (0.27-fold [0.12–0.50]) in deaths from metabolic risks, with the highest ASMR of 5.54 [3.21–8.21]. Tuberculosis caused by high FPG accounted for the highest number of deaths in the low-middle-SDI region, while multidrug-resistant tuberculosis without extensive drug resistance due high FPG showed the most rapid increase in low-SDI region (30.66 [12.94–73.72]). In terms of injuries, deaths caused by metabolic risks were highest in the middle-SDI region. The low-middle-SDI region had the most significant increase (1.42-fold [1.12–1.79]) in injuries from metabolic risks, with the highest ASMR in 2019 at 9.23 (7.57–10.68). Unintentional injuries resulting from low BMD accounted for the highest number of deaths in the middle-SDI region, totaling 83,665 cases [57,454–99,398], while falls caused by low BMD showed the fastest increase (1.90 [1.39–2.54]) (Figure 2).

#### Analysis from the GBD regions

Among the GBD regions worldwide, in NCDs, East Asia, South Asia, and Southeast Asia had the highest number of deaths due to metabolic risks, while Central Asia had the highest ASMR in 2019, with a rate of 514.6 [461.5–566.8]. In East Asia, cardiovascular deaths due to high SBP were predominant, totaling 2.5 million [2.1–3.0].

In 2019, East Asia had the highest DALYs for NCDs due to metabolic risks (93,557,225 [79,955,173–108,500,098]), specifically, the highest number of stroke deaths mainly due to high SBP (25, 958,915 [21,039,885–30,999,709]), corresponding to a high ASDR of 1,271.49 (1,028.97–1,514.34). Central Asia had the largest increase in ASDR of liver cancer due to high FPG with a corresponding EAPC of 4.71 (4.00–5.72) during 1990–2019.

South Asia had the highest number of tuberculosis deaths caused by high FPG among CMNNDs. For injuries, South Asia had the highest number of deaths from unintentional injuries (mainly falls) caused by low BMD, totaling 100,066 [79,381–118,541] deaths. Regions such as Central Asia, Oceania, and South Asia experienced significant increases in deaths due to multidrug-resistant tuberculosis without extensive drug resistance caused by high FPG, with increases of 114.43 (35.56–466.98), 112.57 (22.94–637.49), and 73.14 (12.01–469.26), respectively. High-income North America witnessed a notable decline, with a decrease of −0.71 (−0.89 to −0.33) (Figure 3).

#### Analysis from 204 countries

China, India, and the United States were the top three countries with the highest number of deaths due to metabolic factors in NCDs. In China, the major risk factors contributing to these deaths were high SBP, high low-density lipoprotein (LDL) cholesterol, high FPG, and high body mass index. Uzbekistan, Nauru, and Solomon Islands had the highest ASMR for NCDs due to metabolic risks, with rates of 817.9 (699.4–934.7), 786.9 (671.7–914.0), and 768.3 (630.3–904.3), respectively (Figure 4). In 2019, China and India had the highest number of DALYs cases for NCDs due to metabolic risks. In China, the highest number of stroke cases resulting from high SBP was 25,176,256 [20,271,928–30,148,436], with a corresponding ASDR of 1,279.82 (1,028.18–1,531.38). In India, high SBP led to the highest number of IHD cases, with 19,469,511 [15,743,338–23,437,584] cases, and a corresponding ASDR of 1,673.28 (1,350.93–2,013.57). From 1990 to 2019, Ethiopia had the fastest-growing ASDR for breast cancer deaths due to high body mass index, corresponding to the highest EAPC.

Among CMNNDs, high FPG was the leading metabolic risk factor in all countries, and India had the highest number of deaths attributed to this factor, with 56,421 (34,514–82,627) deaths. The country with the highest ASMR for CMNNDs was the Central African Republic, with the highest ASMR for drug-susceptible tuberculosis mainly caused by high FPG, at 29.5 (16.7–45.0). In terms of injuries, India, China, and the United States had the highest number of deaths due to metabolic risk factors. India also had the highest ASMR for injuries, with a rate of 13.7 (10.8–16.2). Falls, mainly caused by low BMD, were the leading cause of these deaths, with an ASMR of 11.5 (9.0–13.6) (Figures S4 and S5).

### Projections of deaths from risk factors from 2020 to 2040

#### Environmental/occupational risks

**Figure 5:**
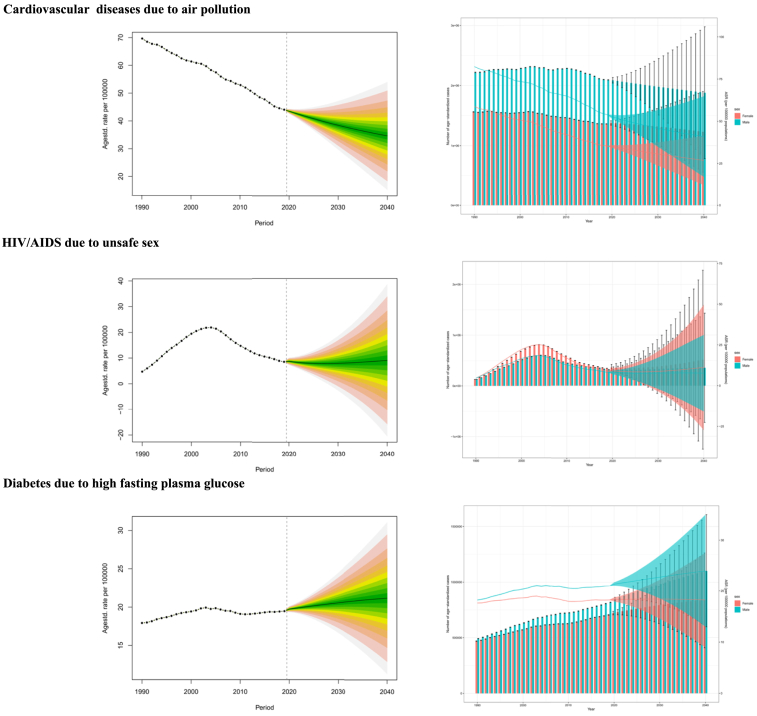
Projections for the number of deaths and ASMR trends related to air pollution-caused cardiovascular disease, HIV/AIDS due to unsafe sex, and diabetes due to high fasting plasma glucose from 2020 to 2040 See also Figures S8–S19.

Among the NCDs due to environmental risks, the primary one is CVD due to air pollution. From 2020 to 2040, the ASMR of CVD attributable to air pollution, while showing a decreasing trend, is projected to remain relatively high at 34.61 in 2040, resulting in a substantial number of deaths, estimated at 4,862,195. When considering gender, male deaths and ASMR significantly exceed females, with projections of 2,685,564 male deaths and 2,176,631 female deaths in 2040. The highest death rate of 483.09 deaths occurs in the 90-to-94 age group (Figures 5 and S8).

Among CMNND due to environmental risks, the ASMR for lower respiratory tract infections due to non-optimal temperatures trends downward from 2020 to 2040, reaching 2.38 in 2040, but still resulting in a high number of deaths at 14,107,325. In terms of gender, both the number of deaths and the ASMR are higher for females than for males by 2040. Regarding the age structure, the age group of 95 years and above has the highest death rate of 1,183.42 (Figures S9 and S10).

From 2020 to 2040, the ASMR for injuries caused by environmental/occupational risks shows a decreasing trend, with an ASMR of only 1.91 and 205,724 deaths in 2040. In terms of gender structure, the number of deaths and ASMR are significantly higher for males than for females. In the age structure, the highest death rate is found in the age group 25–29 years, with a rate of 4.57 (Figure S11).

#### Behavioral risks

Among NCDs due to behavioral risks, CVDs due to dietary risks are more common. The ASMR of CVD due to non-optimal temperatures tends to decrease from 2020 to 2040 but remains high at 73.81, with 11,051,779 deaths in 2040. In terms of gender, both the number of deaths and ASMR are higher in men than in women, with projections of 5,000,998 deaths in women and 6,050,781 deaths in men by 2040. In terms of age structure, the highest mortality rate of 3,268.37 is found among people aged 95 years and above (Figures S12 and S13).

Among CMNNDs resulting from behavioral risks, HIV/AIDS due to unsafe sex is a significant concern, with a slight increase in the ASMR from 2020 to 2040, reaching 9.04 in 2040, and a total of 4,377,319 deaths. Regarding gender, females have higher number of deaths and ASMR compared to males, with projections of 29,443,318 female deaths and 143,304 male deaths in 2040. In terms of age, the highest death rate occurs in the 25–29 age group, at 138.81 (Figures 5 and S14).

The ASMR for injuries due to behavioral risks tends to decrease by 2040, reaching an ASMR of 3.78 with 462,032 deaths. In different gender structures, the number of deaths and the mortality rate are significantly higher among men than among women (projected deaths among men: 388,831; among women: 73,294). In the age structure, the highest mortality rate (49.58) is found among people over 95 years of age (Figures S12 and S15).

#### Metabolic risks

Diabetes due to high FPG constitutes a significant portion of metabolic risk-related NCDs. The ASMR increases from 2020 to 2040, peaking at 21.16 in 2040, resulting in 3,069,093 deaths. Regarding gender, men experience higher number of deaths and ASMR, with projections of 1,577,537 deaths in men and 1,491,556 deaths in women by 2040. In terms of age, the highest death rate is observed in those aged 95 years or older, at 645.34 (Figures 5 and S16,).

Among CMNNDs due to metabolic risks, there is a decreasing trend in ASMR for respiratory infections and tuberculosis due to high FPG from 2020 to 2040, with ASMR reaching 1.24 in 2040 and 174,974 deaths. In terms of gender, both the number of deaths and ASMR are higher in males than in females (projected deaths for females: 45,988, compared to 128,986 for males). In terms of age structure, the highest mortality rate is found in the 85–89-year-old cohort, at 138.81 (Figures S17 and S18).

From 2020 to 2040, the ASMR for injuries due to metabolic risks tends to decrease, with an ASMR of 4.71 and a high number of 742,137 deaths in 2040. In terms of gender, the number of deaths and ASMR are significantly higher for women than for men (projected deaths for women: 417,768, compared to 323,842 deaths for men). In terms of the age structure, the highest mortality rate is found in the population aged 95 years and over, with a death rate of 413.97 (Figures S17 and S19).

## Discussion

### General findings

This comprehensive study provides a thorough analysis of the global burden of 369 disease and injuries associated with environmental/occupational risks, behavioral risks, and metabolic risks. The analysis of global deaths between 1990 and 2019 demonstrates a concerning upward trajectory in deaths caused by behavioral and metabolic risks, while deaths attributed to environmental/occupational risks experienced a slight decline. Despite this decline, environmental/occupational risks remained a significant contributor to global fatalities in 2019. Notably, the swiftest increase in deaths related to NCDs primarily resulted from metabolic risks, while deaths from CMNNDs were predominantly driven by behavioral risks. Injuries were primarily linked to metabolic risks. The projected trends from 2020 to 2040 indicate an upward trend in deaths attributed to environmental/occupational risks and behavioral risks in NCDs, while showing a decline in deaths for CMNNDs and injuries. Metabolic risks contribute to the increasing number of deaths in both NCDs and injuries. Overall, the study emphasizes the urgent need for targeted interventions and policies to address these critical risk factors, mitigate their adverse effects, and alleviate the burden of disease worldwide.

### Environmental/occupational risks

The study reveals that air pollution and non-optimal temperature, especially PM pollution, have emerged as the leading environmental/occupational risks for deaths related to NCDs, including CVD, chronic respiratory diseases, neoplasms, and type 2 diabetes. These risks also contribute to CMNNDs, particularly respiratory infections and tuberculosis. Prolonged exposure to PM induces detrimental physiological changes, including inflammation, oxidative stress, endothelial dysfunction, and lipid peroxidation.^7^^,^^8^ These processes contribute to the progression of atherosclerosis and CVD. Urgent measures are required to address air pollution comprehensively, encompassing systemic changes such as stricter emission regulations and cleaner energy alternatives.

Additionally, non-optimal temperatures (predominantly high temperatures) significantly contribute to deaths from self-harm, interpersonal violence, and motorcyclist road injuries. High temperatures have physiological impacts that exacerbate pre-existing mental health issues, heighten emotional responses, lower impulse control, and potentially increase agitation and aggression.^9^ As for motorcyclist road injuries, high temperatures can also lead to heat-related fatigue, impaired motor function, and cognitive deficits in motorcyclists, increasing the risk of road accidents.^10^^,^^11^ Additionally, discomfort from the heat may reduce the use of protective clothing, exacerbating injury severity. This highlights the need to enhance occupational safety, develop heatwave preparedness strategies, and implement interventions to address the psychological and behavioral consequences of extreme heat events.

Unsafe water and sanitation significantly contribute to mortality from CMNNDs, specifically enteric and respiratory infections. Contaminated drinking water from sewage and agricultural runoff, along with improper storage and treatment methods, is a common cause of diarrheal diseases.^12^^,^^13^ Furthermore, inadequate sanitation facilities contribute to environmental and water source contamination.^13^ Fecal-oral transmission is a major pathway for the spread of intestinal infections caused by pathogens like *Salmonella* and *rotavirus*.^14^ These challenges are particularly prevalent in low- and middle-income regions with limited access to clean water and sanitation facilities. Addressing these issues requires comprehensive approaches, including improving water access, sanitation infrastructure, hygiene education, and effective handwashing practices to prevent intestinal infections and diarrheal diseases.

Occupational risks play a significant role in injury-related deaths, especially in transport-related accidents. Occupations that involve extended working hours, stress, and deadlines increase the likelihood of driver errors and accidents.^15^ To mitigate these risks, workplace safety measures such as implementing safe driving policies, managing fatigue, and reducing stress are crucial. Training in defensive driving, promoting vehicle maintenance, and enforcing anti-distracted driving policies are essential for reducing fatalities.^16^ These findings underscore the importance of implementing occupational safety regulations to prevent workplace accidents and protect workers' health.

### Behavioral risks

This analysis found that tobacco, dietary risks, and alcohol use were the major behavioral risk factors contributing to disease burden globally. Tobacco smoking leads to CVD, neoplasms, and chronic respiratory diseases. It damages blood vessels, promoting cholesterol buildup and plaque formation, thereby increasing the risk of CVD and stroke, while its carcinogens damage DNA, leading to abnormal cell growth and tumor formation.^17^^,^^18^ Similarly, specific associations were observed between dietary risks and CVD, as well as certain types of cancer. Dietary risks, such as high sodium intake, low consumption of fruits and whole grains, and high intake of red meat and trans fatty acids, significantly contribute to CVD. Colon and rectum cancer deaths were associated with diets low in whole grains, milk, and calcium. Alcohol use contributes to cirrhosis and other liver diseases. Excessive alcohol intake results in hepatocyte fat accumulation, stellate cell activation, fibrosis, impaired protein synthesis, and reduced hepatocyte detoxification capacity.^19^ Alcohol use has also been identified as a major risk factor for deaths from self-harm and interpersonal violence particularly among females, underscoring the need for alcohol harm reduction strategies and measures to prevent alcohol-related violence.

Child and maternal malnutrition and unsafe sex are significant contributors to CMNNDs-related deaths. Maternal and child malnutrition primarily leads to neonatal disorders, diarrheal diseases, and mortality related to protein-energy malnutrition. Both genders are susceptible to neonatal disorders due to low birth weight and shortened gestation. Addressing malnutrition is crucial for reducing infant mortality and improving maternal health. Moreover, unsafe sexual practices increase mortality rates associated with HIV/AIDS and sexually transmitted infections. Thus, comprehensive public health measures are crucial, including promoting better dietary habits, enforcing accurate food labeling, enhancing access to nutritious food, advocating safe sexual practices, and implementing alcohol regulatory measures like taxation and restrictions.

### Metabolic risks

Metabolic risks contribute significantly to the disease burden, notably as the primary driver of the rapid increase in deaths related to NCDs and injuries. These metabolic risks mainly encompass factors such as high SBP, high FPG, and low BMD. Hypertension, a highly prevalent and causal risk factor, acts as both a trigger and consequence for CVD and CKD.^20^ The analysis highlighted that high SBP accounted for a substantial proportion of deaths related to CVD and CKD. This is confirmed by the study that high blood pressure stands out among CVD risk factors, presenting the strongest evidence for causation and widespread exposure.^21^^,^^22^ Elevated SBP leads to endothelial dysfunction, vascular constriction, and atherosclerosis, while also causing myocardial hypertrophy, impairing cardiac pumping efficiency, and resulting in ischemic events.^23^^,^^24^ This intricate association is reflected by the pervasive occurrence of hypertension in various stages of CKD and the dual advantages of efficacious antihypertensive therapies in curbing cardio-renal risks.

Diabetes stands as a prominent contributor to global mortality and disability.^25^^,^^26^ High FPG is strongly linked to respiratory infections and tuberculosis in CMNNDs. Chronic hyperglycemia weakens the immune response, damages the respiratory epithelium, disrupts the lung microbiome, and impairs mucociliary clearance, thereby increasing vulnerability to infections.^27^^,^^28^ Managing diabetes through lifestyle modifications and appropriate medical interventions could potentially reduce the risk of respiratory infections in individuals with CMNNDs.

Osteoporosis is becoming increasingly prominent with the aging population.^29^ Low BMD is a significant risk factor for fatal falls, particularly among older individuals. The strong association between low BMD and increased fracture risk, especially in the hip, and spine, has been well documented.^30^ Notably, mortality rates related to falls associated with low BMD have shown substantial growth from 1990 to 2019. These alarming trends highlight the urgent need for proactive measures to prevent falls and improve bone health, particularly among older individuals. Early identification of individuals with low BMD through bone density screenings is of paramount importance. Lifestyle modifications, such as ensuring calcium and vitamin D intake, and adopting fall-prevention strategies, should be pursued.^31^

### Regional and country variations

The analysis also reveals significant regional and county variations. For example, low-SDI regions experienced the highest increase in NCD-related deaths, primarily due to unsafe water, sanitation, handwashing, and injuries. Middle-SDI regions were notable for deaths related to dietary risks and tobacco-related CVD. High-SDI regions experienced a remarkable increase in deaths from cocaine use disorders. East Asia and South Asia emerged as high-risk areas for NCDs, particularly CVDs caused by air pollution and dietary risks, while South Asia and Western Sub-Saharan Africa faced significant challenges from CMNNDs, including enteric infections and neonatal disorders. Injuries associated with environmental/occupational risks were prominent in South and East Asia. The analysis across 204 countries highlighted the enormous burden faced by populous nations such as China and India in terms of NCD-related deaths. Additionally, behavioral risks played a significant role in driving NCDs, with tobacco being prominent in China and dietary risks in India.

The variation in risk factors and disease burden across regions underscores the importance of targeted strategies that address local challenges. For instance, efforts to improve water and sanitation infrastructure are crucial in low-SDI regions, while air pollution reduction measures are needed in East Asia and South Asia. Comprehensive prevention and control measures for behavioral risk factors such as tobacco and alcohol use are necessary globally. Strengthening healthcare systems, enhancing disease surveillance, and improving access to preventive measures and treatment are vital components of the response. The findings also highlight the need for international collaboration and resource allocation to effectively tackle the burden of diseases. Overall, the study emphasizes significant regional variations in disease burden, highlighting the need for tailored interventions and preventive measures based on regional contexts and needs.

### Implications and limitations

The findings of this study carry significant implications for global health policy. Efforts to reduce environmental/occupational risks should focus on curbing air pollution, improving temperature regulation, and ensuring workplace safety. Addressing behavioral risks requires comprehensive tobacco control measures, promoting healthy diets, and tackling alcohol and drug misuse. Mitigating metabolic risks should prioritize strategies focusing on blood pressure, glucose control, and osteoporosis prevention. The study underscores the importance of a multifaceted approach involving collaboration between governments, public health organizations, and communities to effectively tackle these risk factors.

### Conclusion

The comprehensive study provides a compelling analysis of the global, regional, and national burden of 369 diseases and injuries attributable to environmental/occupational risks, behavioral risks, and metabolic risks from 1990 to 2019. The study highlights that the burden of diseases continues to be significantly impacted, with NCDs mainly due to metabolic risks, CMNNDs due to behavioral risks, and injuries due to metabolic risks.

Such insights emphasize the importance of integrated, cross-sectoral strategies to mitigate these risk factors and lower the disease load. By implementing evidence-based interventions and adopting a holistic approach, policymakers can make substantial progress in alleviating the global burden of disease and improving population health outcomes.

### Limitations of the study

This study is subject to certain limitations. Firstly, GBD data collection relies on various sources across different countries, including national health surveys, medical records, and registries, which may introduce selection bias. Secondly, variations in data quality and availability among countries can result in differences in the accuracy and completeness of the information used in the study. The projection analysis fails to consider the potential effects of the COVID-19 pandemic on disease deaths and burden due to the unavailability of relevant data at the time. Lastly, the absence of longitudinal studies in altered databases hinders the complete determination of causality and the direction of relationships.

## STAR★Methods

### Key resources table

**Table:** 

REAGENT or RESOURCE	SOURCE	IDENTIFIER
**Software and algorithms**		

Prism 9.0	GraphPad Software	https://www.graphpad.com
R version 4.1.2	The R Foundation for Statistical Computing	https://www.r-project.org/

**Other**		

GBD 2019	IHME	https://www.healthdata.org/

### Resource availability

#### Lead contact

Further information and requests for resources and reagents should be directed to and will be fulfilled by the lead contact, Xiaodong Sun (xiaodong.sun@wfmc.edu.cn).

#### Materials availability

This study did not generate new unique reagents.

#### Data and code availability

• All data reported in this paper will be shared by the lead contact upon request.
• This paper does not report original code.
• Any additional information required to reanalyze the data reported in this paper is available from the lead contact upon request.

### Experimental model and study participant details

This study did not generate experimental model or enroll subjects.

### Method details

#### Overview and data collection

This study relied upon data from the GBD Study 2019, a comprehensive and standardized endeavor that integrates diverse data sources to estimate the worldwide burden of diseases and injuries.

#### Study population

The study population encompassed individuals of all ages and both sexes worldwide. The burden of 369 diseases and injuries was estimated for 204 countries and territories, allowing for analysis at the global, regional, and national levels.

#### Disease and injury classification

To ensure uniformity and comparability across different locations and timeframes, a standardized classification system was adopted for diseases and injuries. Specifically, the International Classification of Diseases (ICD) system, particularly ICD-10, was employed for coding and categorizing these health conditions. Diseases and injuries were classified into five levels by the GBD Working Group.

#### Risk factors

The study encompassed a wide array of risk factors associated with the occurrence and progression of diseases and injuries. These risk factors span across diverse domains, including environmental/occupational, behavioral, and metabolic factors.

#### Study design

The study conducted analyses across various regions, countries, genders, and age groups (0-14 years, 15-49 years, 50-69 years, and 70+ years). Additionally, the study also explores the relationship between exposure and socio-demographic development. The Socio-demographic Index (SDI) is a composite indicator used to measure the socio-demographic development status of a region, consisting of data on per capita income, education level, and fertility rate; it also serves as a standard for measuring exposure. This examination provided insights into the interconnection between the economic environment and risk factors. This study complies with the Guidelines for Accurate and Transparent Health Estimates Reporting recommendations.

### Quantification and statistical analysis

The study quantified the contribution of each risk factor to the burden of disease and injury using an attributable score approach. This approach involved estimating the proportion of the disease burden attributable to specific risk factors, considering their prevalence and relative risk. Statistical modeling techniques, such as Bayesian meta-regression analysis, were employed to estimate the burden of disease and injury in the GBD study. Additionally, uncertainty intervals, defined by lower and upper bounds based on the 2.5th and 97.5th percentiles, were generated to account for the inherent variability and uncertainty within the data. Finally, we conducted BAPC analysis in R with the assistance of software packages such as BAPC (version 0.0.36). This enabled us to project ASR by gender and the number of deaths from 2019 to 2040. All data analysis was carried out using the open-source software R (version 4.1.2).^32^^,^^33^

## Supplemental information

Document S1. Figures S1–S19

Table S1. Key diseases attributable to environmental/occupational, behavioral, and metabolic risks in 2019, related to Figure 1

Table S2. Global deaths attributable to environmental/occupational, behavioral, and metabolic risks from 1990 to 2019, related to Figure 1

## References

[1] GBD 2019 Diseases and Injuries Collaborators (2020). Global burden of 369 diseases and injuries in 204 countries and territories, 1990-2019: a systematic analysis for the Global Burden of Disease Study 2019. Lancet.

[2] Lancet T. (2022). Non-communicable diseases: what now. Lancet.

[3] Emadi M., Delavari S., Bayati M. (2021). Global socioeconomic inequality in the burden of communicable and non-communicable diseases and injuries: an analysis on global burden of disease study 2019. BMC Publ. Health.

[4] Baker R.E., Mahmud A.S., Miller I.F., Rajeev M., Rasambainarivo F., Rice B.L., Takahashi S., Tatem A.J., Wagner C.E., Wang L.F. (2022). Infectious disease in an era of global change. Nat. Rev. Microbiol..

[5] GBD 2017 Risk Factor Collaborators (2018). Global, regional, and national comparative risk assessment of 84 behavioural, environmental and occupational, and metabolic risks or clusters of risks for 195 countries and territories, 1990-2017: a systematic analysis for the Global Burden of Disease Study 2017. Lancet.

[6] Sokolow S.H., Nova N., Jones I.J., Wood C.L., Lafferty K.D., Garchitorena A., Hopkins S.R., Lund A.J., MacDonald A.J., LeBoa C. (2022). Ecological and socioeconomic factors associated with the human burden of environmentally mediated pathogens: a global analysis. Lancet Planet. Health.

[7] Arias-Pérez R.D., Taborda N.A., Gómez D.M., Narvaez J.F., Porras J., Hernandez J.C. (2020). Inflammatory effects of particulate matter air pollution. Environ. Sci. Pollut. Res. Int..

[8] Macchi C., Sirtori C.R., Corsini A., Mannuccio Mannucci P., Ruscica M. (2023). Pollution from fine particulate matter and atherosclerosis: A narrative review. Environ. Int..

[9] Li D., Zhang Y., Li X., Zhang K., Lu Y., Brown R.D. (2023). Climatic and meteorological exposure and mental and behavioral health: A systematic review and meta-analysis. Sci. Total Environ..

[10] Hou K., Zhang L., Xu X., Yang F., Chen B., Hu W. (2022). Ambient temperatures associated with increased risk of motor vehicle crashes in New York and Chicago. Sci. Total Environ..

[11] Liang M., Zhao D., Wu Y., Ye P., Wang Y., Yao Z., Bi P., Duan L., Sun Y. (2021). Short-term effects of ambient temperature and road traffic accident injuries in Dalian, Northern China: A distributed lag non-linear analysis. Accid. Anal. Prev..

[12] Mahmud Z.H., Islam M.S., Imran K.M., Hakim S.A.I., Worth M., Ahmed A., Hossan S., Haider M., Islam M.R., Hossain F. (2019). Occurrence of Escherichia coli and faecal coliforms in drinking water at source and household point-of-use in Rohingya camps, Bangladesh. Gut Pathog..

[13] Wolf J., Hunter P.R., Freeman M.C., Cumming O., Clasen T., Bartram J., Higgins J.P.T., Johnston R., Medlicott K., Boisson S., Prüss-Ustün A. (2018). Impact of drinking water, sanitation and handwashing with soap on childhood diarrhoeal disease: updated meta-analysis and meta-regression. Trop. Med. Int. Health.

[14] Overgaard H.J., Dada N., Lenhart A., Stenström T.A.B., Alexander N. (2021). Integrated disease management: arboviral infections and waterborne diarrhoea. Bull. World Health Organ..

[15] Soccolich S., Ridgeway C., Mabry J.E., Camden M.C., Miller A., Iridiastadi H., Hanowski R.J. (2022). Challenges in Conducting Empirical Epidemiological Research with Truck and Bus Drivers in Diverse Settings in North America. Int. J. Environ. Res. Publ. Health.

[16] Mueller A.S., Cicchino J.B., Zuby D.S. (2020). What humanlike errors do autonomous vehicles need to avoid to maximize safety. J. Saf. Res..

[17] Benowitz N.L., Liakoni E. (2022). Tobacco use disorder and cardiovascular health. Addiction.

[18] Tang M.S., Lee H.W., Weng M.W., Wang H.T., Hu Y., Chen L.C., Park S.H., Chan H.W., Xu J., Wu X.R. (2022). DNA damage, DNA repair and carcinogenicity: Tobacco smoke versus electronic cigarette aerosol. Mutat. Res. Rev. Mutat. Res..

[19] Namachivayam A., Valsala Gopalakrishnan A. (2021). A review on molecular mechanism of alcoholic liver disease. Life Sci..

[20] Pugh D., Gallacher P.J., Dhaun N. (2019). Management of Hypertension in Chronic Kidney Disease. Drugs.

[21] Fuchs F.D., Whelton P.K. (2020). High Blood Pressure and Cardiovascular Disease. Hypertension.

[22] Burnier M., Damianaki A. (2023). Hypertension as Cardiovascular Risk Factor in Chronic Kidney Disease. Circ. Res..

[23] Bryan N.S. (2022). Nitric oxide deficiency is a primary driver of hypertension. Biochem. Pharmacol..

[24] Ishikawa Y. (2004). [High blood pressure and cardiac hypertrophy]. Nihon Rinsho..

[25] GBD 2021 Diabetes Collaborators (2023). Global, regional, and national burden of diabetes from 1990 to 2021, with projections of prevalence to 2050: a systematic analysis for the Global Burden of Disease Study 2021. Lancet.

[26] Zhang K., Kan C., Han F., Zhang J., Ding C., Guo Z., Huang N., Zhang Y., Hou N., Sun X. (2023). Global, Regional, and National Epidemiology of Diabetes in Children From 1990 to 2019. JAMA Pediatr..

[27] Baker E.H., Baines D.L. (2018). Airway Glucose Homeostasis: A New Target in the Prevention and Treatment of Pulmonary Infection. Chest.

[28] Proença de Oliveira-Maul J., Barbosa de Carvalho H., Goto D.M., Maia R.M., Fló C., Barnabé V., Franco D.R., Benabou S., Perracini M.R., Jacob-Filho W. (2013). Aging, diabetes, and hypertension are associated with decreased nasal mucociliary clearance. Chest.

[29] Khandelwal S., Lane N.E. (2023). Osteoporosis: Review of Etiology, Mechanisms, and Approach to Management in the Aging Population. Endocrinol. Metab. Clin. North Am..

[30] Zhang X., Xu H., Li G.H., Long M.T., Cheung C.L., Vasan R.S., Hsu Y.H., Kiel D.P., Liu C.T. (2021). Metabolomics Insights into Osteoporosis Through Association With Bone Mineral Density. J. Bone Miner. Res..

[31] Wei F.L., Li T., Gao Q.Y., Huang Y., Zhou C.P., Wang W., Qian J.X. (2022). Association Between Vitamin D Supplementation and Fall Prevention. Front. Endocrinol..

[32] Riebler A., Held L. (2017). Projecting the future burden of cancer: Bayesian age-period-cohort analysis with integrated nested Laplace approximations. Biom. J..

[33] Liu Z., Xu K., Jiang Y., Cai N., Fan J., Mao X., Suo C., Jin L., Zhang T., Chen X. (2021). Global trend of aetiology-based primary liver cancer incidence from 1990 to 2030: a modelling study. Int. J. Epidemiol..

