# Supplementary material for: Epidemiology of 369 diseases and injuries attributable to 84 risk factors: 1990–2019 with 2040 projection
Source: iScience. 2024 Mar 14;27(4):109508. doi: 10.1016/j.isci.2024.109508 (PMC11075060; doi:10.1016/j.isci.2024.109508)

iScience, Volume *27*

Supplemental information

Epidemiology of 369 diseases and injuries

attributable to 84 risk

factors: 1990–2019 with 2040 projection

Kexin Zhang, Chengxia Kan, Jian Chen, Junfeng Shi, Yanhui Ma, Xiaoli Wang, Xuan Li, Weiqin Cai, Ruiyan Pan, Jingwen Zhang, Zhentao Guo, Fang Han, Ningning Hou, and Xiaodong Sun

Supplementary figures

Figure S1: Gender effects: all-risk factors, environmental/occupational risks, behavioral risks, and metabolic risks induced deaths from NCDs, CMNNDs, injurie, and total cancer, related to Figure 1.

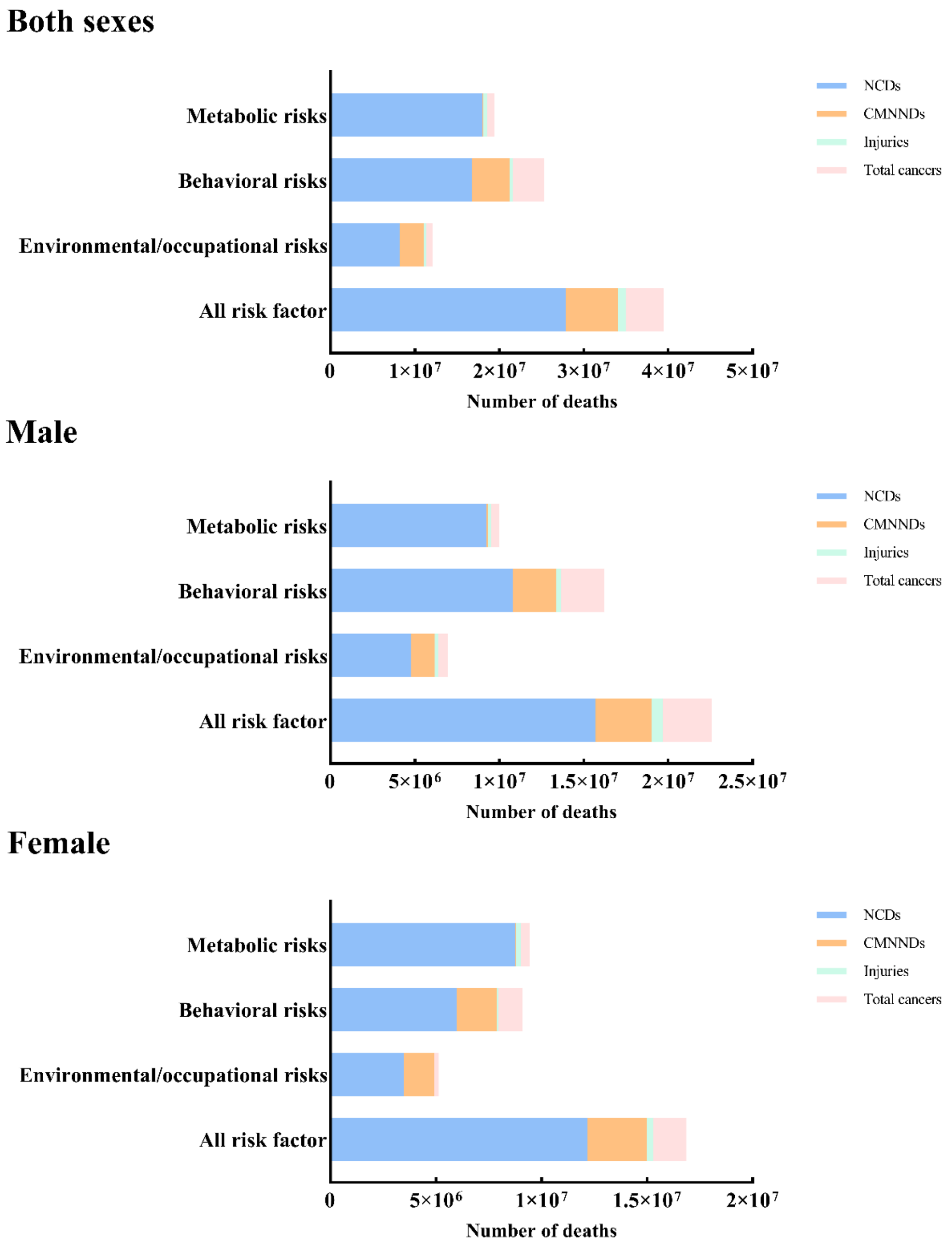

Figure S2: The number of deaths in NCDs due to different environmental/occupational risks, behavioral risks, and metabolic risks in four different age subgroups: 0-14 years, 15-49 years, 50-69 years, and 70+ years, related to Figure 1.

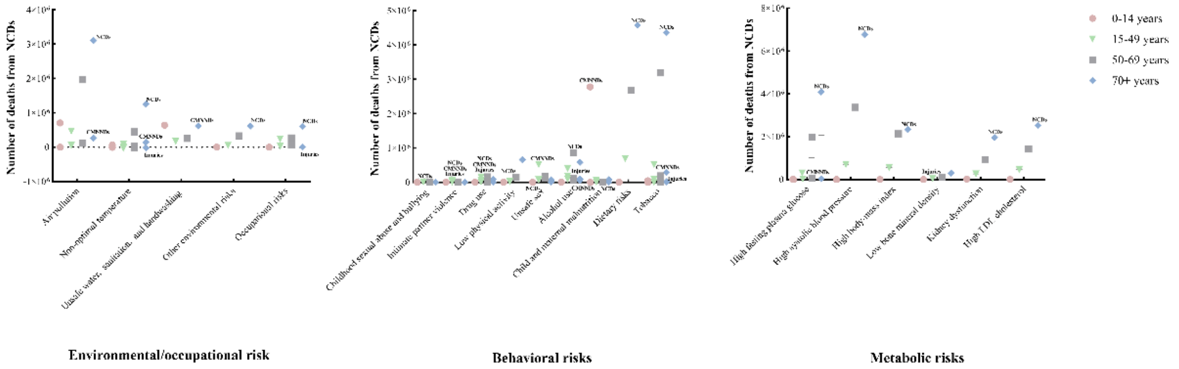

Figure S3: ASMR for NCDs, CMNNDs, injurie due to risk factors such as environmental/occupational risks, behavioral risks, and metabolic risks, related to Figure 1.

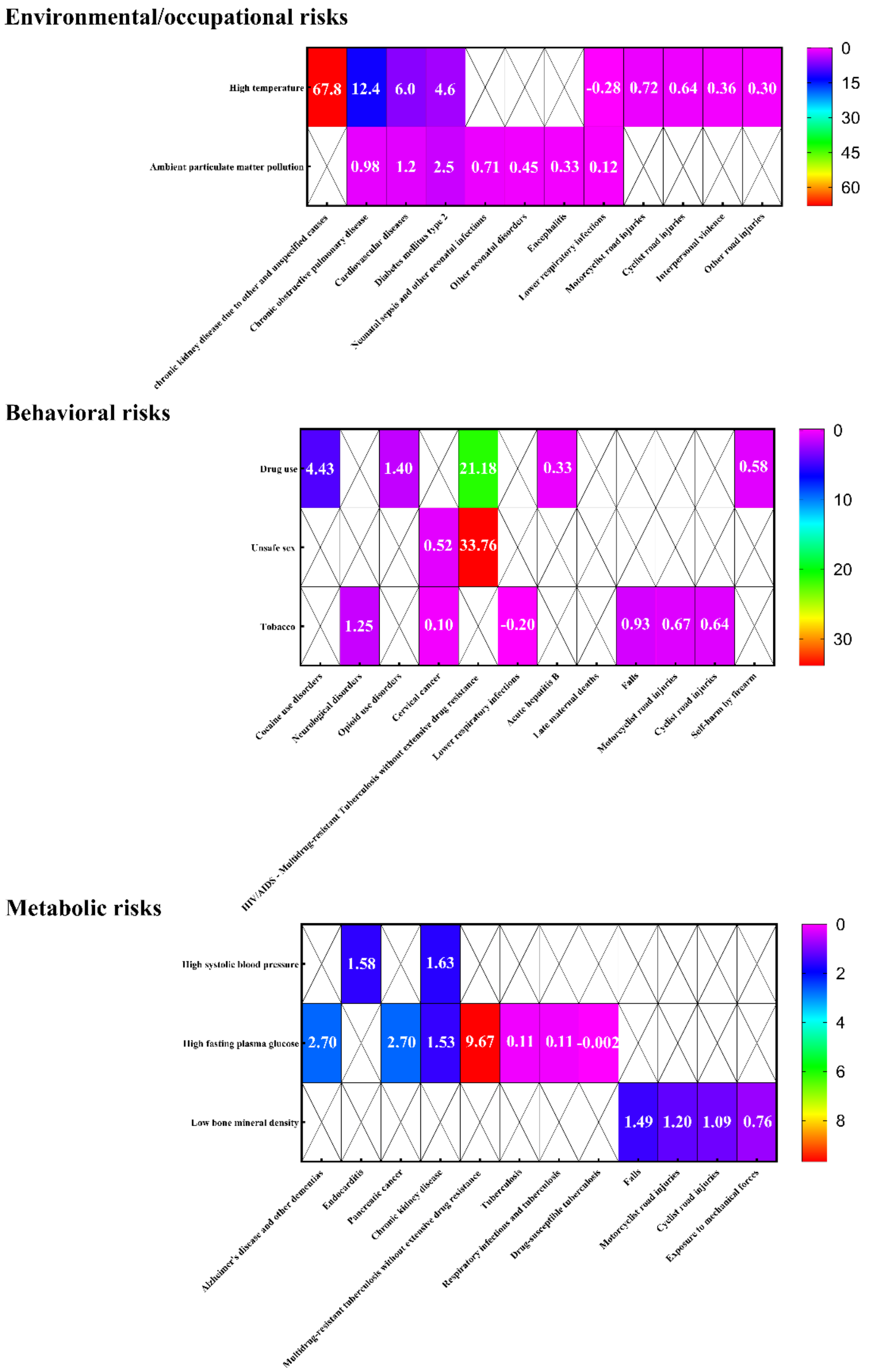

Figure S4. ASMR for CMNND due to risk factors such as environmental/occupational risks, behavioral risks, and metabolic risks in 204 countries, related to Figure 4.

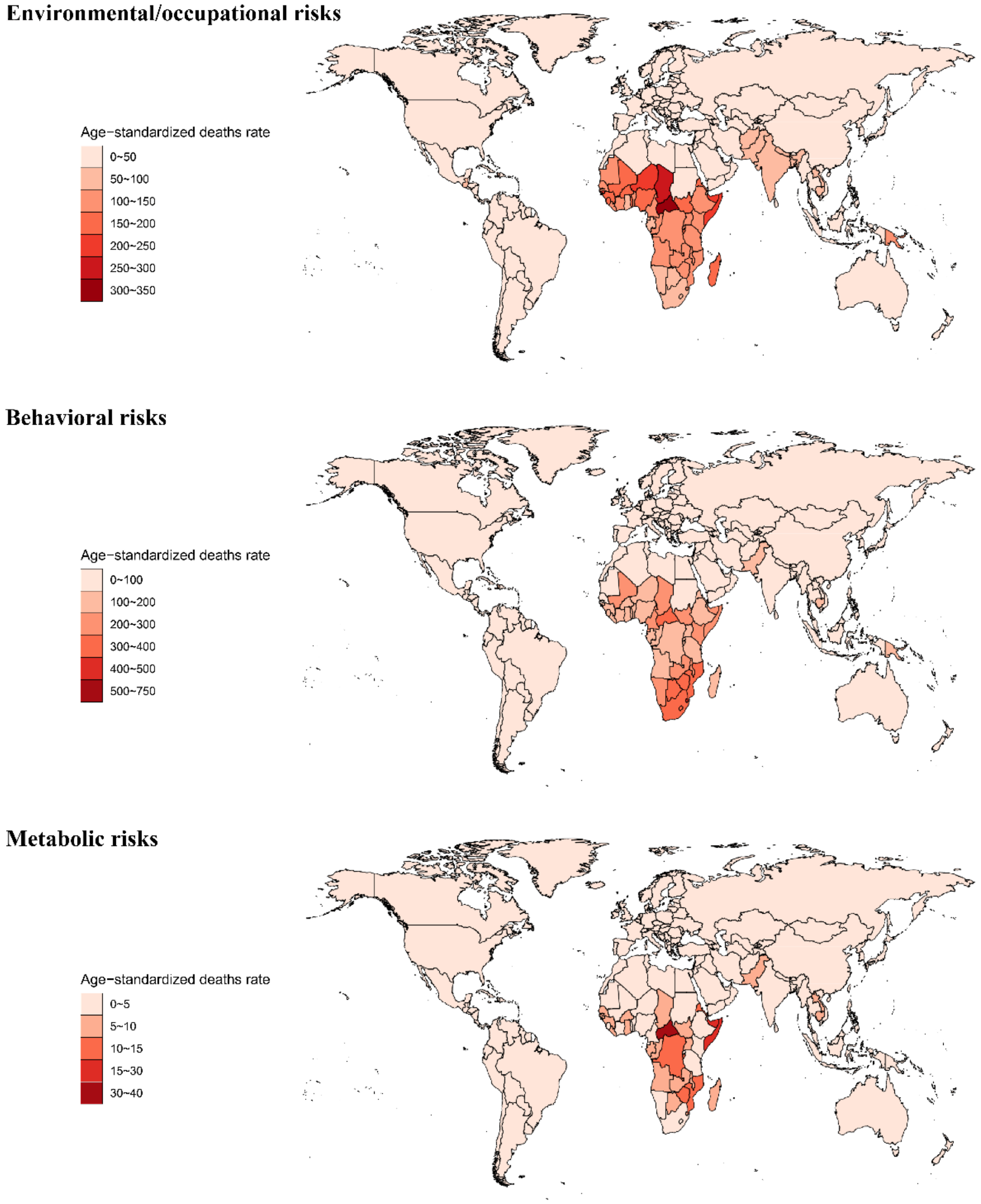

Figure S5. ASMR for injuries due to risk factors such as environmental/occupational risks, behavioral risks, and metabolic risks in 204 countries, related to Figure 4.

Environmental/occupational risks

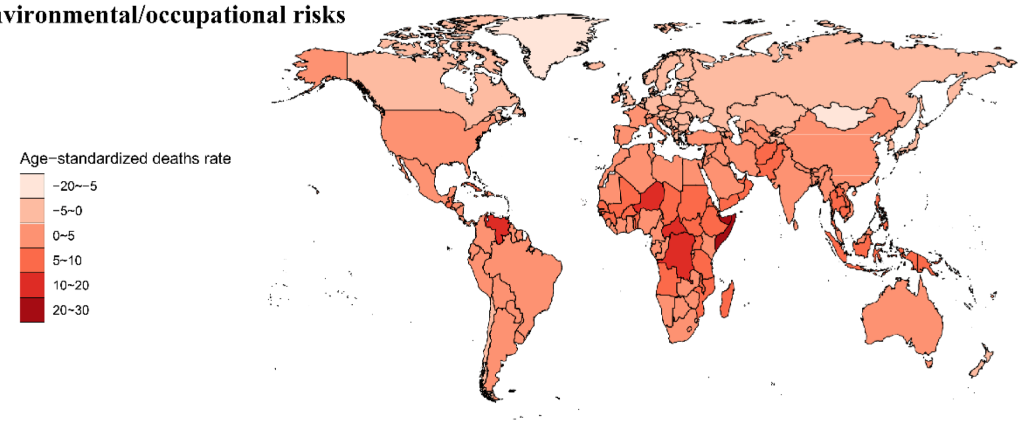

Behavioral risks

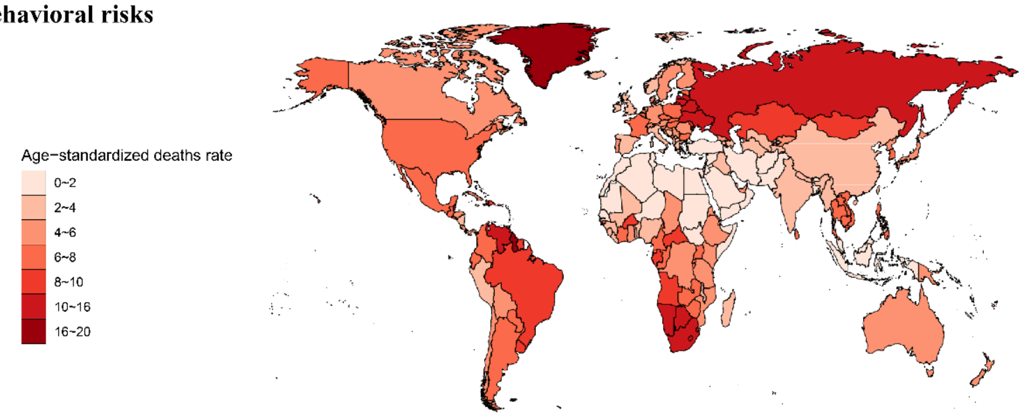

Metabolic risks

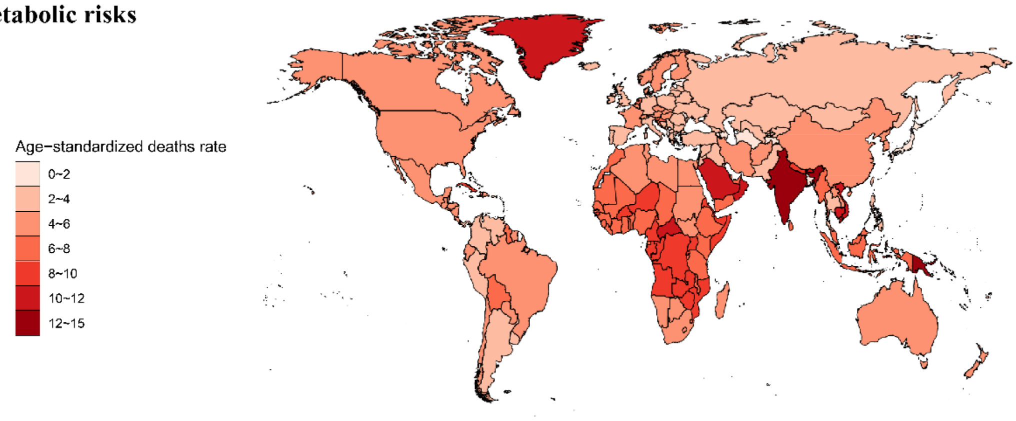

Figure S6. ASMR of NCDs, CMNNDs, injurie attributable to behavioral risk factors, 1990-2019.

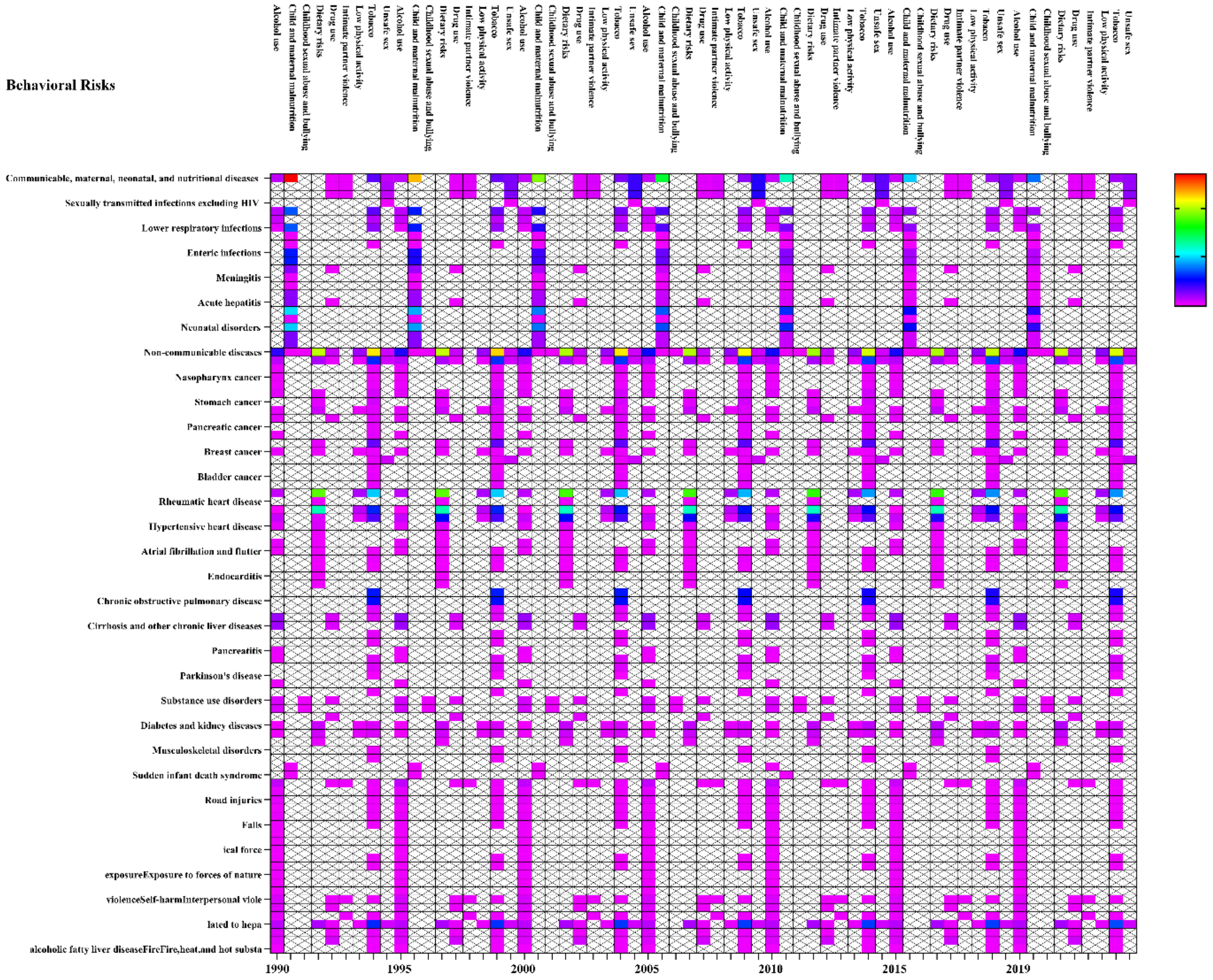

Figure S7. ASMR of NCDs, CMNNDs, injurie attributable to Metabolic risk factors, 1990-2019.

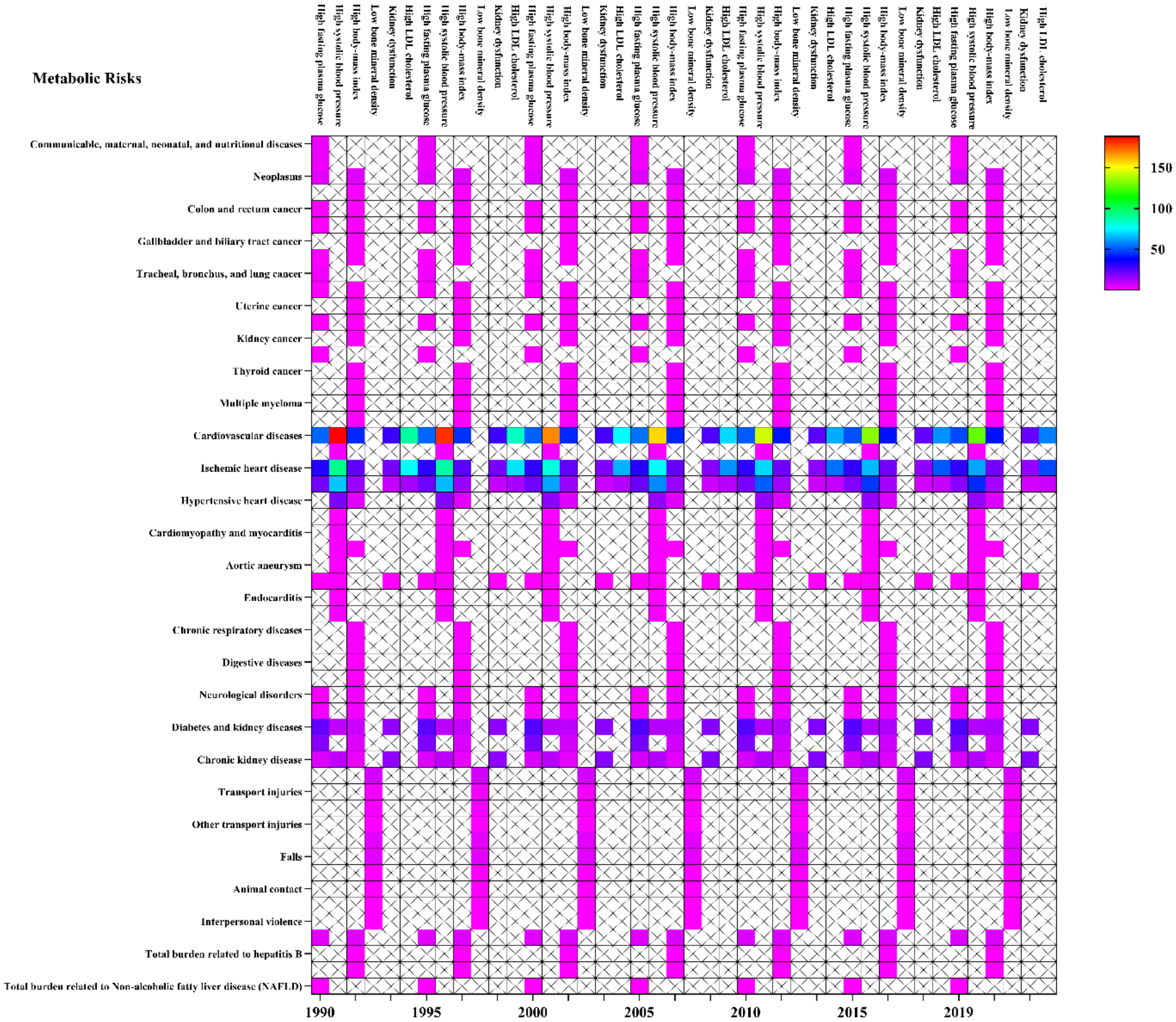

Figure S8: ASMR changes in air pollution due to cardiovascular disease in different age groups from 2020 to 2040, related to Figure 5.

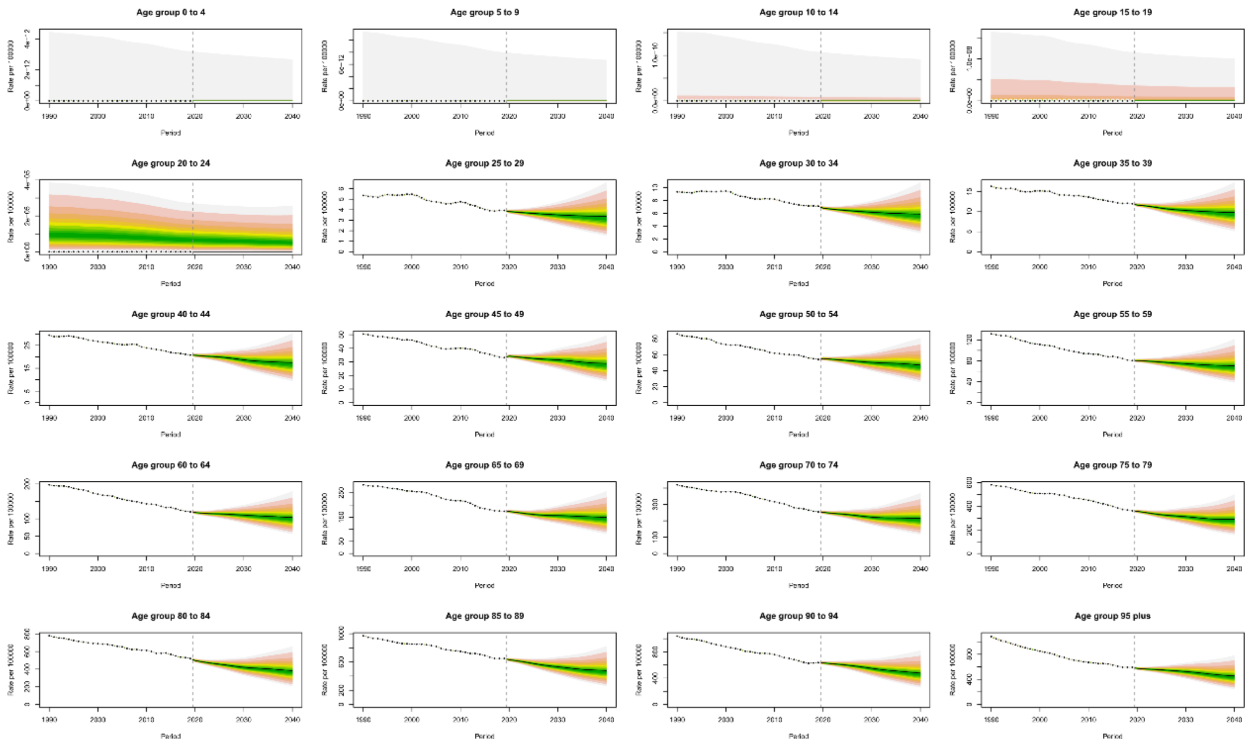

Figure S9: ASMR changes in lower respiratory tract infections due to non-optimal temperatures in different age groups from 2020 to 2040, related to Figure 5.

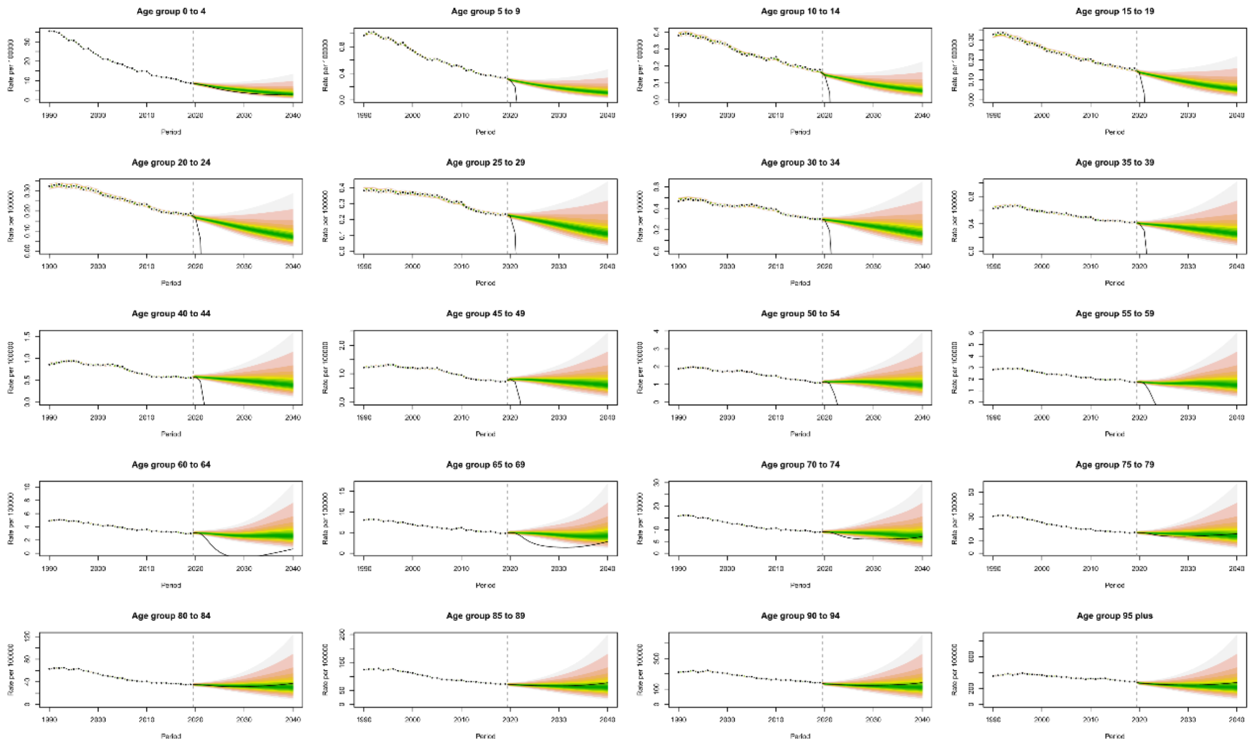

Figure S10: ASMR of CMNND and injuries due to environmental risks, and direct sex-specific mortality and death rates, related to Figure 5.

Lower respiratory tract infections due to non-optimal temperatures

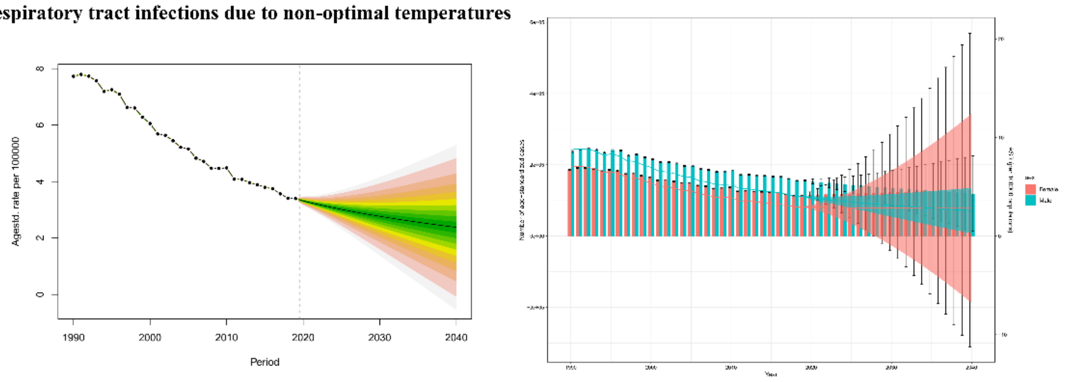

Injuries caused by environmental/occupational risks

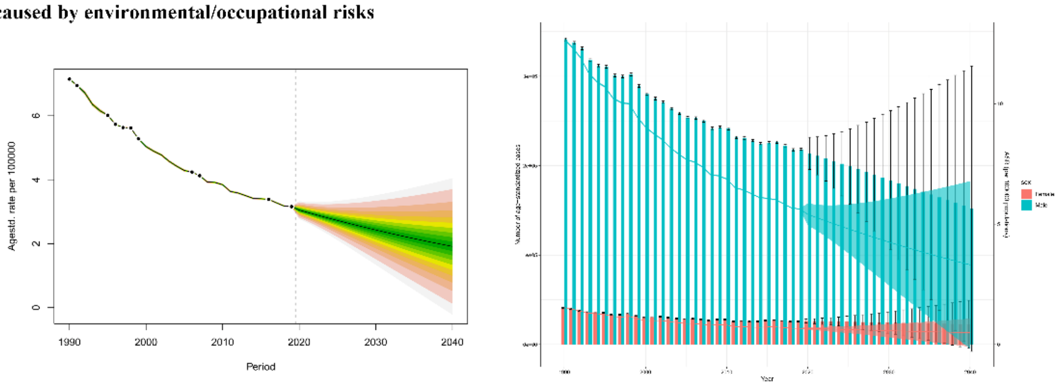

Figure S11: ASMR changes in injuries due to environmental/occupational risks in different age groups from 2020 to 2040, related to Figure 5.

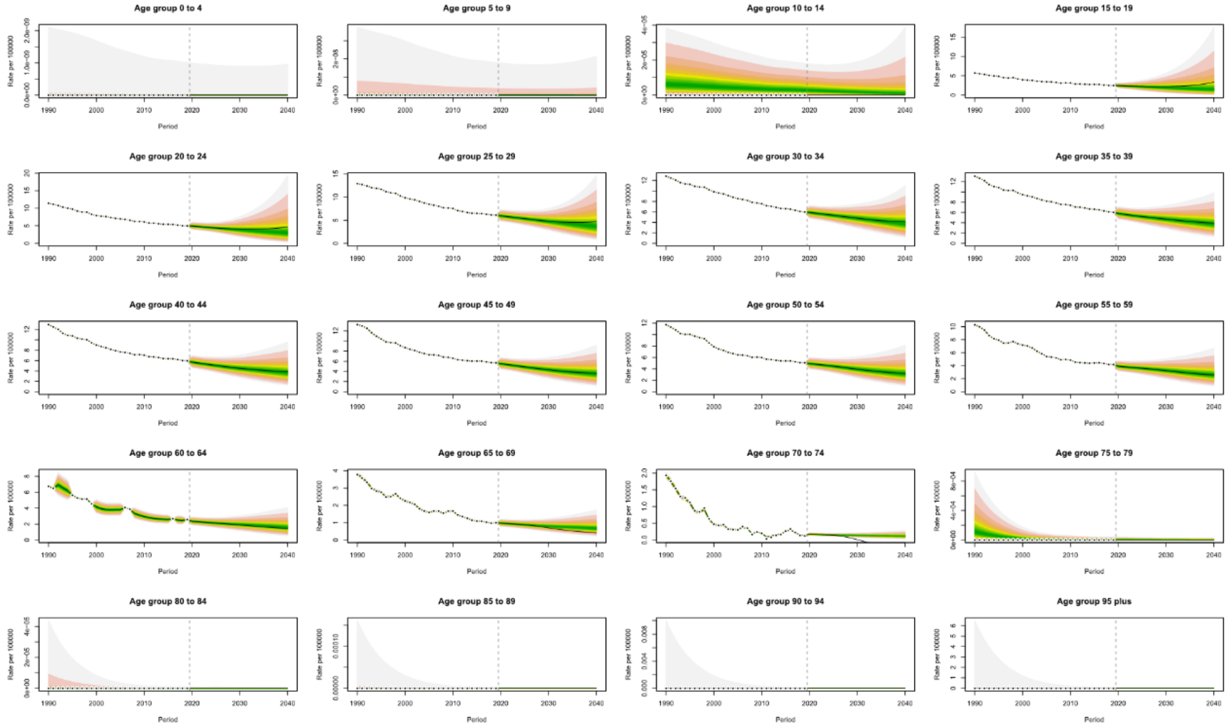

Figure S12: ASMR of NCD and injuries due to behavioral risks, and direct sex-specific mortality and death rates, related to Figure 5.

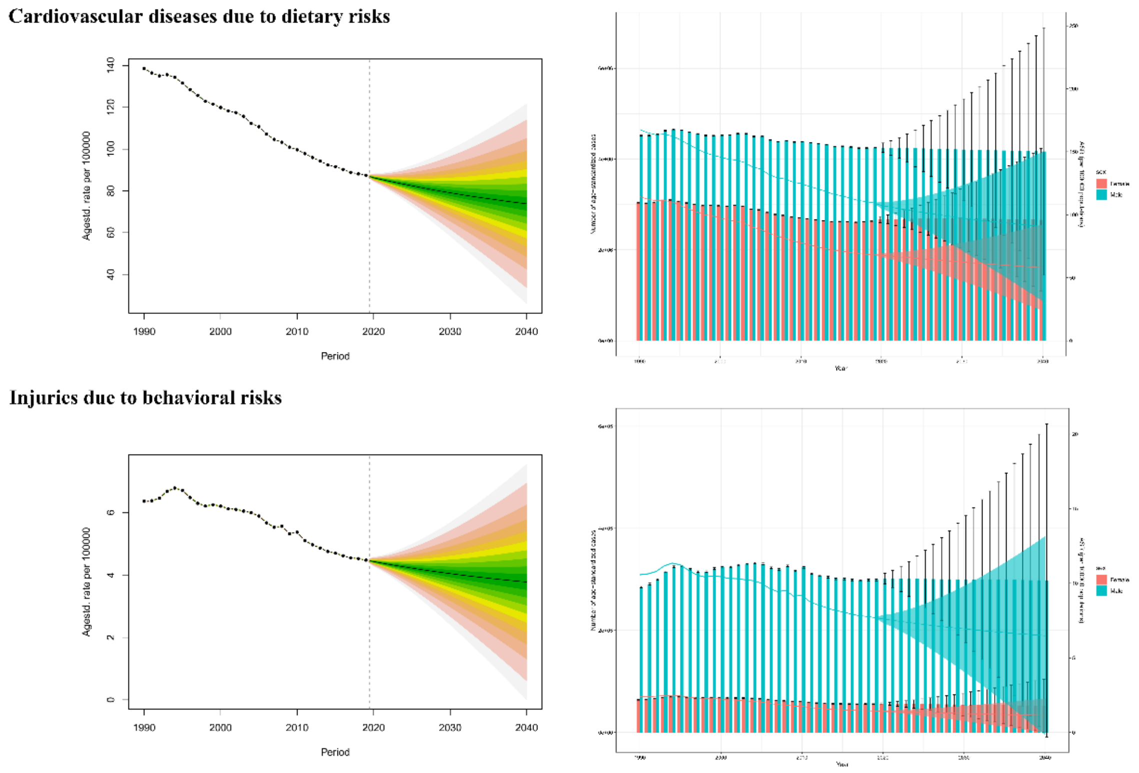

Figure S13: ASMR changes in cardiovascular diseases due to dietary risks in different age groups from 2020 to 2040, related to Figure 5.

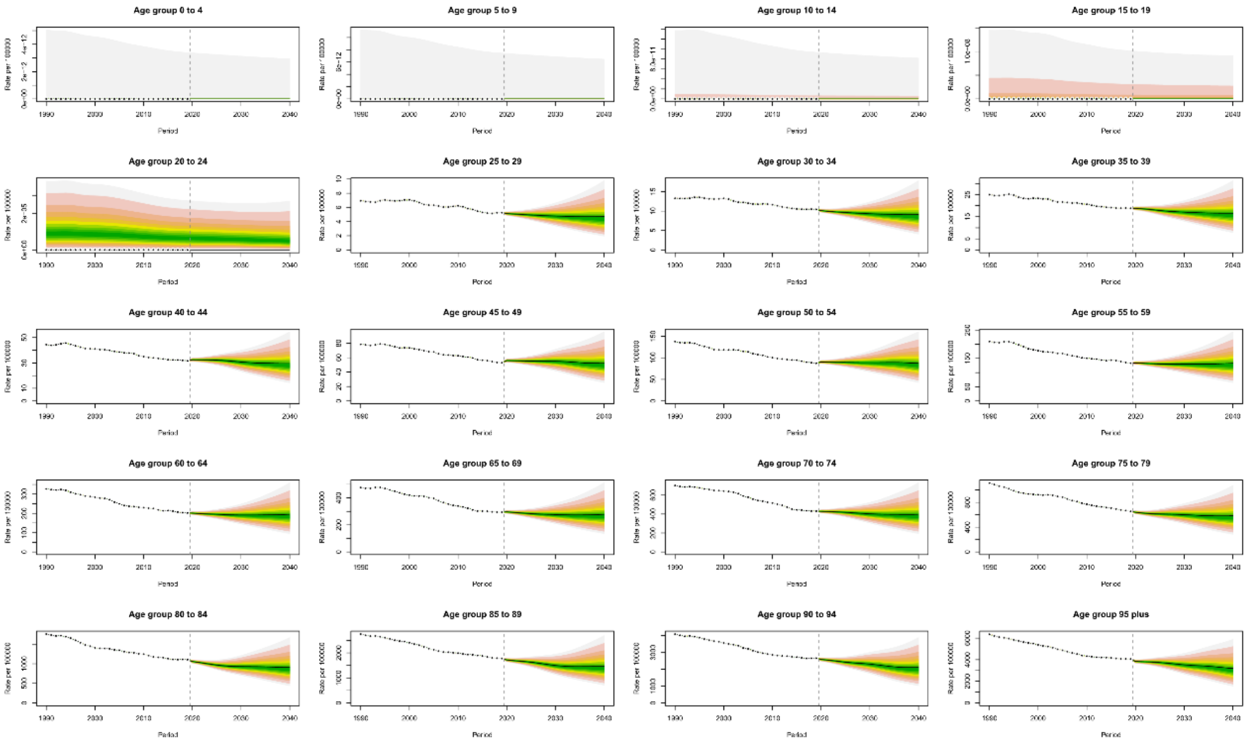

Figure S14: ASMR changes in HIV/AIDS due to unsafe sex in different age groups from 2020 to 2040, related to Figure 5.

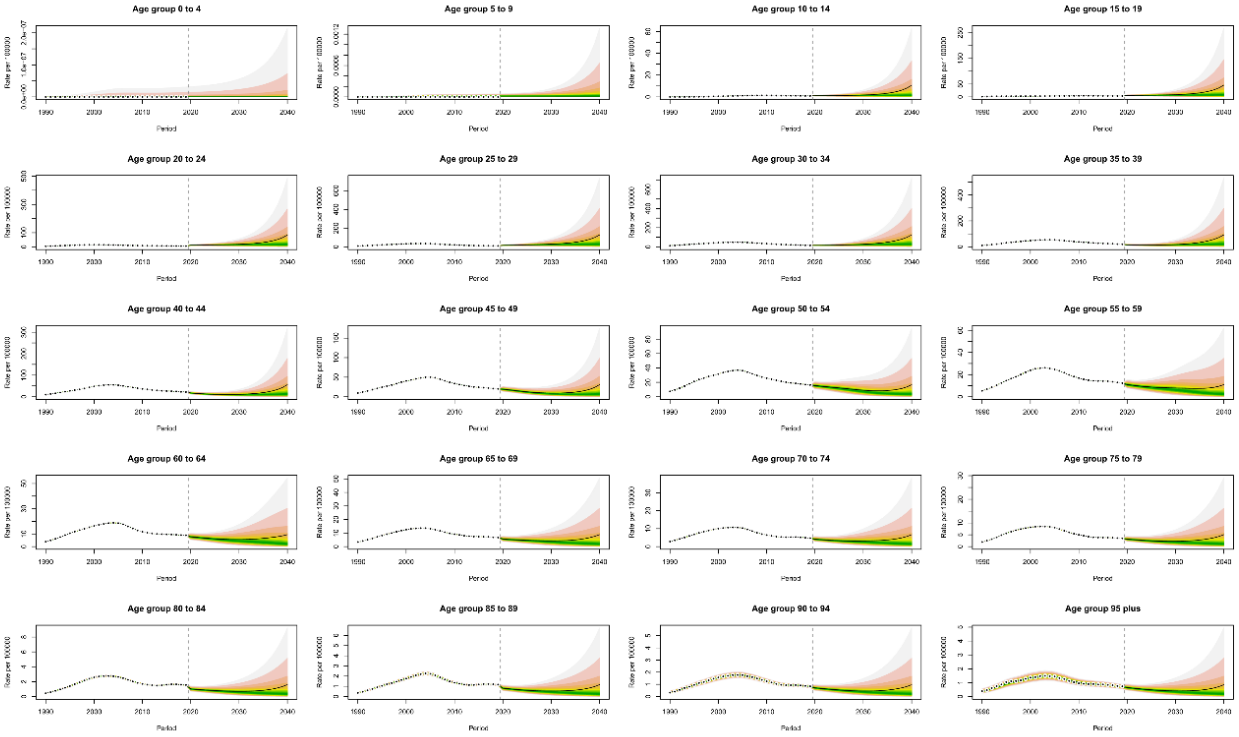

Figure S15: ASMR changes in injuries due to behavioral risks in different age groups from 2020 to 2040, related to Figure 5.

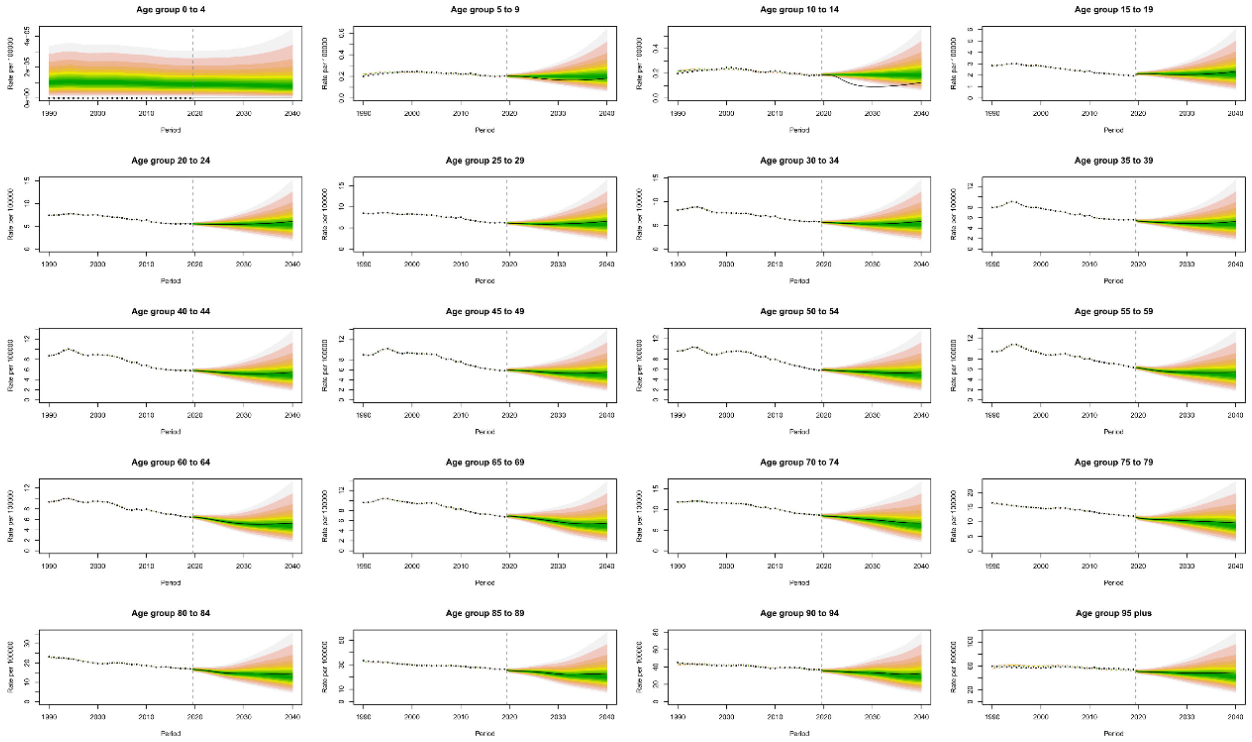

Figure S16: ASMR changes in diabetes due to high fasting plasma glucose in different age groups from 2020 to 2040, related to Figure 5.

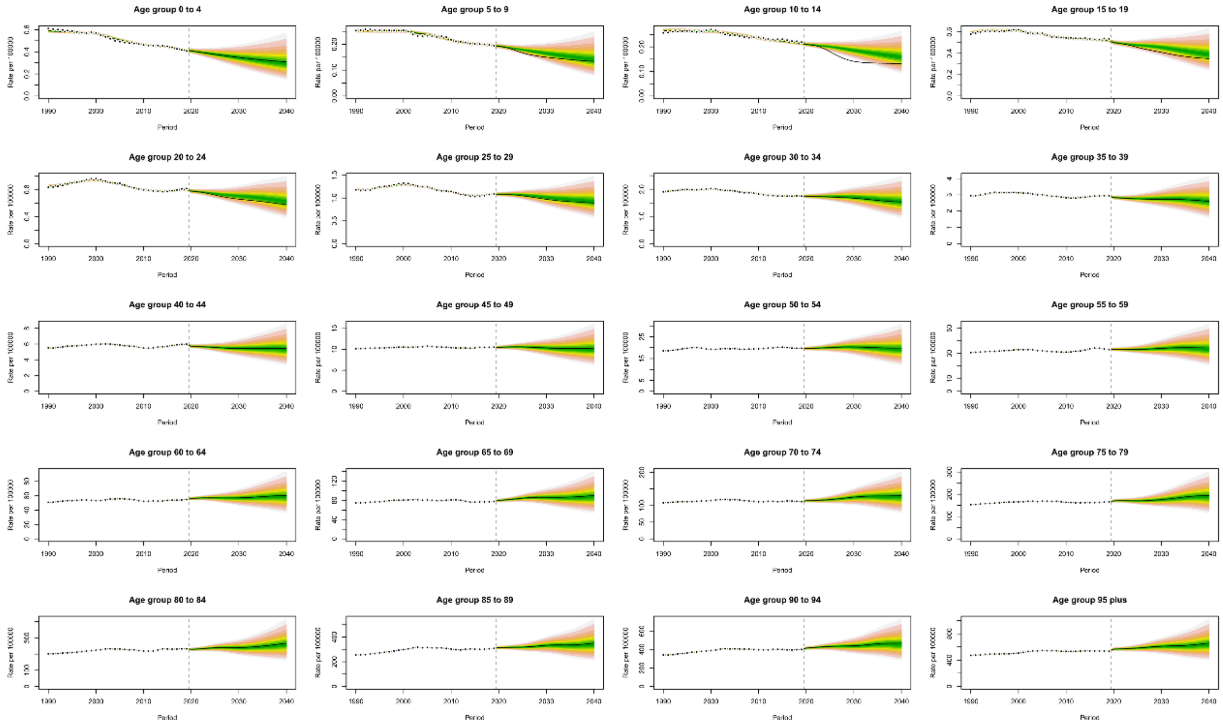

Figure S17: ASMR of CMNND and injuries due to metabolic risks, and direct sex-specific mortality and death rates, related to Figure 5.

Respiratory infections and tuberculosis due to high fasting plasma glucose

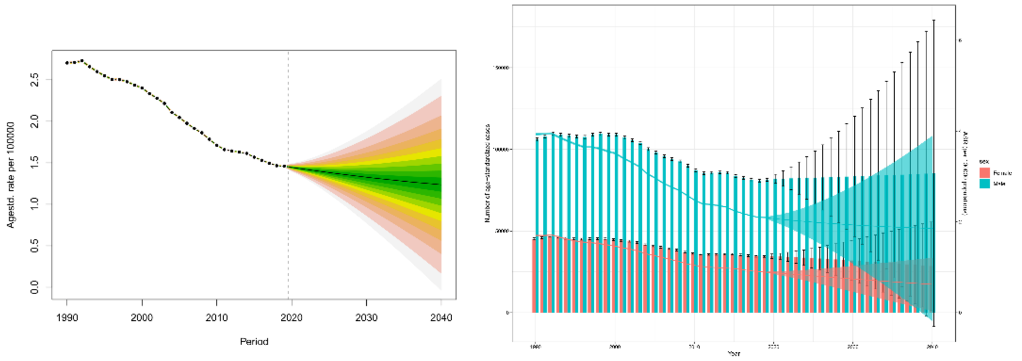

Injuries due to metabolic risks

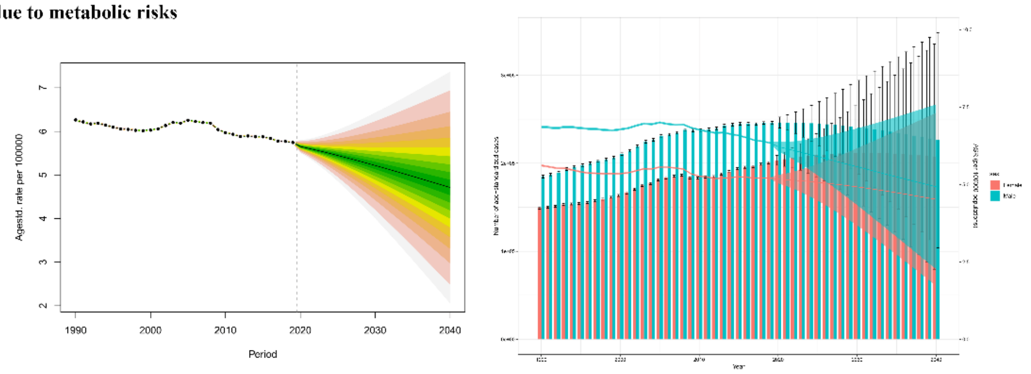

Figure S18: ASMR changes in respiratory infections and tuberculosis due to high fasting plasma glucose in different age groups from 2020 to 2040, related to Figure 5.

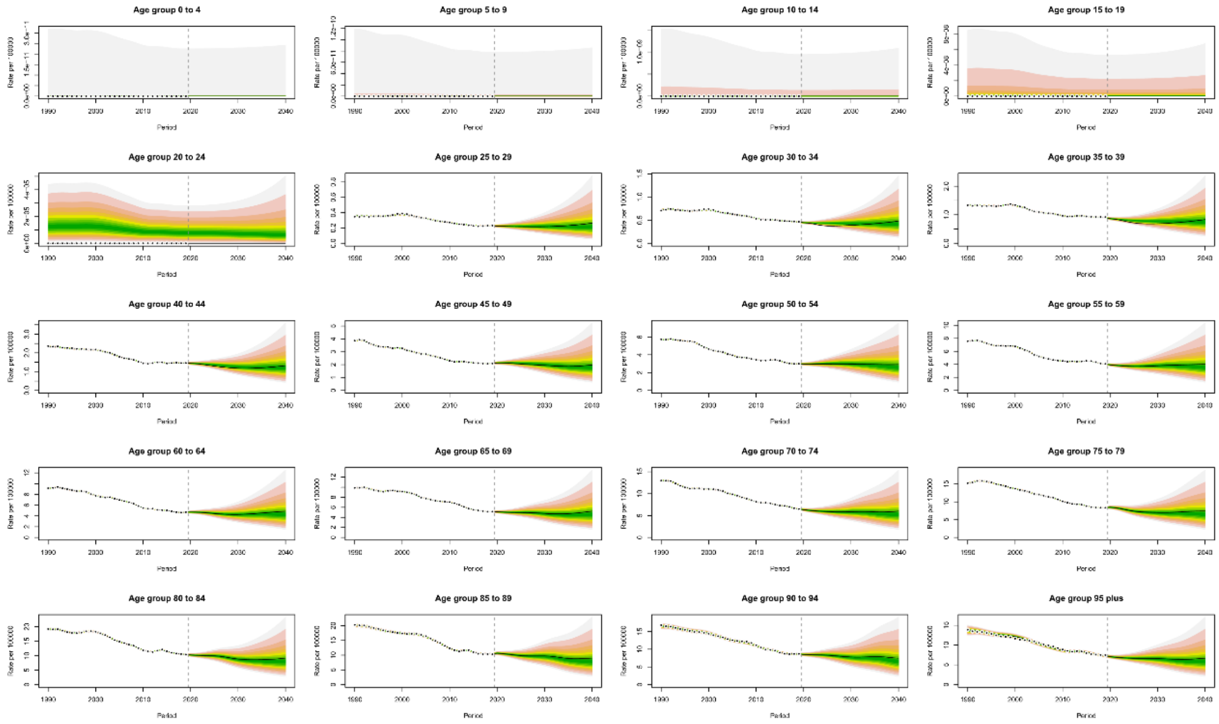

Figure S19: ASMR changes in injuries due to metabolic risks in different age groups from 2020 to 2040, related to Figure 5.

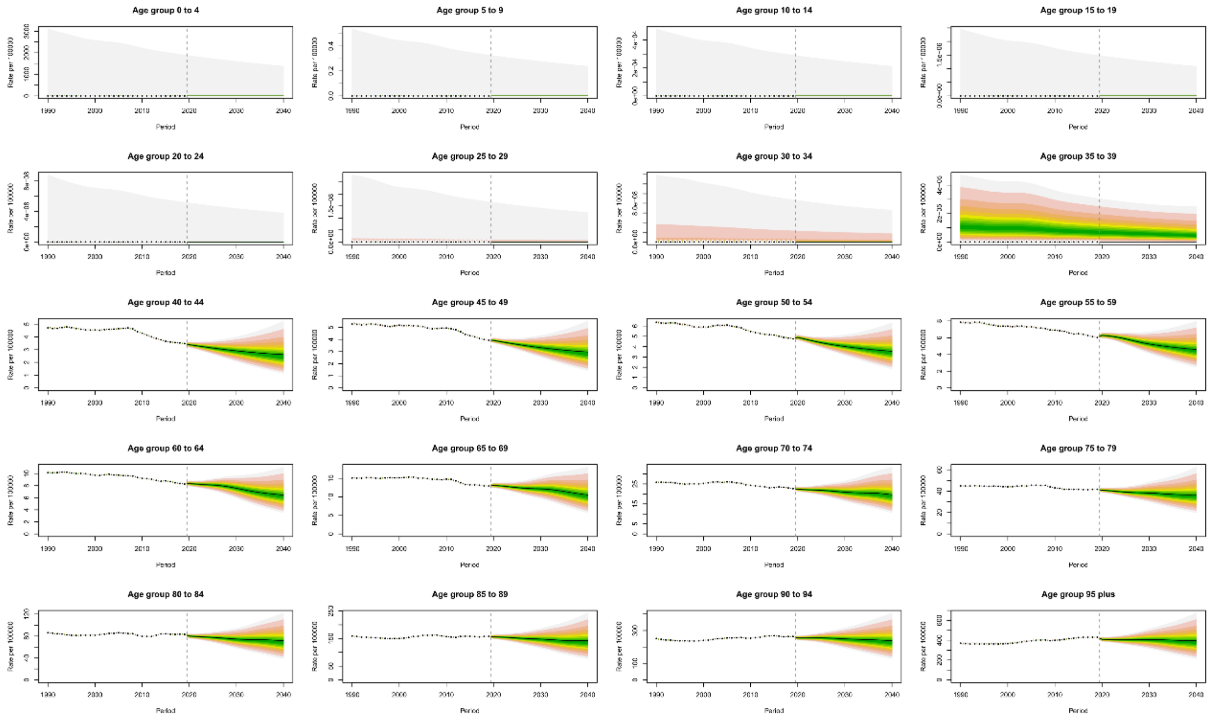

Supplement: Document S1. Figures S1–S19 [file mmc1.pdf]
